# Glucose Uncouples Nitrogen Sensing From Chlorosis via a Photosynthetic Checkpoint in *Synechocystis* sp. PCC 6803

**DOI:** 10.1111/ppl.70645

**Published:** 2025-11-21

**Authors:** Pablo Ortega‐Martínez, Joaquín Giner‐Lamia, Laura T. Wey, M. Isabel Muro‐Pastor, Francisco J. Florencio, Sandra Díaz‐Troya

**Affiliations:** ^1^ Instituto de Bioquímica Vegetal y Fotosíntesis, Universidad de Sevilla‐Consejo Superior de Investigaciones Científicas Seville Spain; ^2^ Departamento de Bioquímica Vegetal y Biología Molecular, Facultad de Biología Universidad de Sevilla Seville Spain; ^3^ Department of Life Technologies University of Turku Turku Finland

**Keywords:** carbon metabolism, chlorosis, cyanobacteria, nitrogen metabolism, photomixotrophy, photosynthesis

## Abstract

Cyanobacteria adapt to nitrogen starvation by undergoing chlorosis, a regulated bleaching process that involves the degradation of phycobilisomes, the light‐harvesting antennae complexes, and accumulation of glycogen. While this response is well characterized under photoautotrophic conditions, its modulation by external organic carbon sources such as glucose remains poorly characterized. Here, we investigated how glucose affects the response to nitrogen deprivation in the model cyanobacterium *Synechocystis* sp. PCC 6803. Using an integrative approach combining physiological assays, targeted metabolomics, RNA sequencing, chlorophyll fluorescence and absorbance spectroscopy, we studied the underlying regulatory mechanisms, focusing on photosynthetic electron transport. Glucose supplementation prevented bleaching, even when added after nitrogen deprivation symptoms had begun. This effect was associated with excess glycogen accumulation, disrupted carbon partitioning, and buildup of metabolic intermediates, indicating a metabolic overflow. Despite these physiological differences, transcriptomic responses to nitrogen deprivation were largely similar regardless of glucose supplementation, suggesting regulation at the post‐transcriptional or metabolic level. Glucose also impaired photosynthetic electron transport by creating a redox bottleneck at the photosystem II (PSII) acceptor side, leading to decreased electron transport to photosystem I (PSI) and oxidation of the P700 pool. These findings suggest that reduction of the P700 acceptor side is required to trigger chlorosis. Our results demonstrate that glucose uncouples nitrogen sensing from the bleaching process by altering photosynthetic electron flow. We propose the existence of a redox‐sensitive checkpoint that integrates metabolic state with photosynthetic performance, offering new insights into stress adaptation in cyanobacteria.

## 1 Introduction

Cyanobacteria are an ancient and diverse lineage of oxygenic photosynthetic prokaryotes that have shaped Earth's biosphere for over 2.5 billion years (Schirrmeister et al. 2016). Throughout their evolutionary history, they have adapted to a wide range of ecological niches by developing diverse physiological and metabolic acclimation strategies to cope with fluctuating environmental conditions. Some cyanobacterial species, including some sub‐strains of *Synechocystis* sp. PCC 6803 (hereafter *Synechocystis*), possess metabolic flexibility that enables them to grow under photomixotrophic conditions by fixing CO_2_ via photosynthesis while simultaneously assimilating exogenous organic carbon sources such as glucose (Aaron Kaplan et al. 2008; Koskinen et al. 2023).

As a non‐diazotrophic cyanobacterium, *Synechocystis* is unable to fix atmospheric nitrogen (N_2_), and has developed complex regulatory responses to survive the limitation of this essential nutrient. Upon limitation of combined nitrogen forms, such as ammonium (NH_4_^+^), nitrate (NO_3_^−^), or urea, *Synechocystis* undergoes an adaptive program known as chlorosis (Forchhammer and Schwarz 2019). This process involves a profound reorganization of the photosynthetic machinery and cell metabolism, allowing cells to enter a dormant‐like state and survive for long periods of nitrogen starvation.

The response to nitrogen depletion is triggered by an imbalance in C/N metabolism (Forchhammer and Selim 2020). In the absence of nitrogen assimilation, the GS‐GOGAT cycle becomes limited, which leads to an increase in GOGAT's substrate 2‐oxoglutarate (2‐OG), the key C/N sensing metabolite. The accumulation of 2‐OG is detected by the signal transduction protein PII, and the global nitrogen regulator NtcA (Muro‐Pastor et al. 2001; Esteves‐Ferreira et al. 2018; Forchhammer and Schwarz 2019). These proteins coordinate the transcriptomic and metabolic response for the acclimation to nitrogen starvation (Esteves‐Ferreira et al. 2018).

A key manifestation of this response is the degradation of the phycobilisomes (PBS), the light‐harvesting antennae complexes, shifting the cell's blue‐green colouring to orange‐yellow, a process termed bleaching. This is a strategy with a dual function. Firstly, it allows the recycling of the nitrogen present in these large protein complexes as a supply for de novo synthesis of proteins to adapt to this new nutrient situation. Secondly, the decrease in light‐harvesting capacity lowers the excitation pressure on the photosynthetic electron transport chain (PETC), thereby avoiding potential overreduction and photodamage (Salomon et al. 2013; Baier et al. 2014; Levi et al. 2018; Forchhammer and Schwarz 2019). As an outcome, glycogen reserves are highly increased through the carbon from the phycobiliprotein degradation and the fixed CO_2_ (Forchhammer and Schwarz 2019). This is promoted by the redirection of the carbon flux towards gluconeogenic reactions through the inactivation of phosphoglycerate mutase (PGAM). This regulation is caused by the association of PGAM with the small protein CfrA/PirC, an interactor of PII that becomes highly expressed and released from PII under nitrogen deprivation (Muro‐Pastor et al. 2020; Orthwein et al. 2021).

PBS degradation is an orchestrated process involving several components. Among the most relevant are the non‐bleaching (Nbl) proteins NblA, NblB and NblD, whose expression is induced by NtcA (Baier et al. 2014; Forchhammer and Schwarz 2019; Krauspe et al. 2021). In *Synechocystis*, the NblA1/NblA2 heterodimer interacts with the PBS (Sendersky et al. 2015; Nguyen et al. 2017), facilitating pigment disassociation by the NblB bilin lyase (Levi et al. 2018) and PBS degradation by the ATP‐dependent ClpC‐ClpP1‐ClpR protease complex (Karradt et al. 2008; Baier et al. 2014). NblD also plays an important role in PBS degradation, although its precise function remains unclear (Krauspe et al. 2021). The process begins from the phycocyanin rods, progressing to the allophycocyanin core until nearly complete PBS degradation (Sendersky et al. 2015).

Several scenarios prevent the bleaching process, including limitations on cell growth (e.g., carbenicillin and cerulenin) or photosynthesis imposed by light restrictions or inhibitors (e.g., DCMU and DBMIB) (Salomon et al. 2013; Yoshihara and Kobayashi 2022) or the incorporation of 2‐OG through a 2‐OG permease (Hickman et al. 2013). Despite being able to grow photomixotrophically, a previous report has observed bleaching inhibition by glucose supplementation in *Synechocystis* (Elmorjani and Herdman 1987).

Additionally, chlorosis upon nitrogen depletion is hindered in specific mutant strains. As expected, mutants defective in the regulation of the response to nitrogen deprivation (e.g., Δ*ntcA*; Sauer et al. 1999) or in genes required to degrade PBS (Δ*nblA*, Δ*nblB* or Δ*nblD*; Baier et al. 2014; Levi et al. 2018) are unable to perform bleaching. An additional group of mutants that fail to perform bleaching consists of those unable to accumulate glycogen. Mutants lacking ADP‐glucose pyrophosphorylase (AGP), the enzyme catalyzing the first committed step in the synthesis of glycogen, are unable to degrade their phycobiliproteins in response to nitrogen deprivation, despite sensing nitrogen deficiency and activating the corresponding transcriptional program (Gründel et al. 2012; Hickman et al. 2013; Carrieri et al. 2017). Upon nitrogen deprivation, glycogen‐deficient mutants also exhibited growth arrest, metabolic overflow, and a progressive and fast closure of PSII reaction centers, decreasing O_2_ evolution despite retaining PBS complexes (Carrieri et al. 2012; Gründel et al. 2012; Ortega‐Martínez et al. 2023).

Despite extensive study of nitrogen starvation responses, the biochemical mechanism by which glucose supplementation halts the chlorosis program in *Synechocystis* under nitrogen deprivation remains unresolved. Notably, this response is counterintuitive: it might be expected that the increased C/N ratio caused by exogenous glucose would promote chlorosis. Instead, here, we report that the additional carbon input from glucose supplementation results in a metabolic imbalance that impairs photosynthetic electron transport and prevents PBS degradation. Bleaching is blocked even though nitrogen deficiency is perceived by canonical sensors, such as the PII protein, and the NtcA‐regulon is transcriptionally activated. This suggests that additional regulatory layers potentially linked to photosynthetic activity and redox balance modulate the bleaching process. Therefore, managing cellular carbon pools becomes crucial for an adequate response to nitrogen deprivation. Furthermore, our results help explain why chlorosis is suppressed under various conditions that disrupt carbon flux or photosynthesis, including growth‐limiting environments, glycogen metabolism mutants, and low‐light or photosynthesis‐inhibiting treatments.

## 2 Materials and Methods

### 2.1 Strains and Culture Conditions

For routine cell culture, the glucose‐tolerant *Synechocystis* sp. PCC 6803 strains were cultivated under photoautotrophic conditions in BG11 medium, with 17.6 mM NaNO_3_ as the nitrogen source, and supplemented with 12 mM NaHCO_3_ (referred to as BG11C) (Rippka et al. 1979; Ortega‐Martínez et al. 2024). Cultures were grown at 30°C under continuous light (4500 k LED lights, 50 μmol photons m^−2^ s^−1^) in conical flasks bubbled with a stream of 1% (v/v) CO_2_ in air. *Synechocystis* culture growth was monitored by optical density measurement at 750 nm in a UV–Vis GENESYS180 spectrophotometer (ThermoFisher). Whole‐cell absorption spectra were recorded in cultures adjusted to an OD_750_ of one using visible light ranging from 400 to 800 nm. When required, media were supplemented with the required antibiotics (50 μg mL^−1^ nourseothricin).

For nitrogen deprivation, strains were harvested twice by centrifugation (10 min at 7000 ×g) in nitrate‐free BG11C medium (BG11_0_C) and finally resuspended at one OD_750_ in BG11_0_C. Cultures were divided and cultured under control (C) conditions or supplemented with 4 mM glucose (G).

### 2.2 Generation of Mutant Strains

The ΔG6PDH strain lacking the *slr1843* gene was obtained by transformation of the WT strain with plasmid pSPARK_∆G6PDH::Nat(+), which allows for the deletion of the complete *slr1843* ORF. This plasmid contains a nourseothricin‐resistance cassette flanked by sequences 511 pb upstream and 650 pb downstream of the *slr1843* ORF. Complete segregation was confirmed by PCR with primers *slr1843* UP 5′ (5′‐GGTGGGACTATTACCGGG‐3′) and *slr1843* R (5′‐GAGATAGCTTTCTGGG‐3′).

### 2.3 Glycogen Quantification and Glucose Consumption Determination

Glycogen content was determined as in Ortega‐Martínez et al. (2023). Briefly, glucose released from purified glycogen and glucose content in the medium were measured with a glucose oxidase assay, employing standard curves of known concentrations of amyloglucosidase‐digested glycogen and glucose, respectively.

### 2.4 Glutamine Synthetase Activity

Measurements of in situ glutamine synthetase activity were performed as described in Merida et al. (1991) using the Mn^2+^‐dependent γ‐glutamyl‐transferase assay in cells permeabilized with alkyltrimethylammonium bromide (MTA). The concentration of the resulting γ‐glutamylhydroxamic was determined spectrophotometrically at 500 nm using a molar extinction coefficient of 0.89 mM^−1^·cm^−1^. One U of GS activity corresponds to the amount of enzyme required to produce 1 μmol product per minute.

### 2.5 Metabolite Extraction and Targeted Metabolomics

Intracellular metabolites were extracted and determined by LC–MS as described in Ortega‐Martínez et al. (2024). Chromatographic separation was performed with a XSELECT HSS XP 150 mm × 2.1 mm × 2.5 μm (Waters) in an Exion HPLC (Sciex) connected to a QTrap 6500+ (Sciex) operating in negative mode.

### 2.6 RNA Extraction and RNA‐Seq Analysis

Cultures (30 OD_750_) were harvested by centrifugation (10 min, 4°C, 4200× g) and pellets frozen in liquid nitrogen and stored at −70°C. Cell pellets were resuspended in 450 μL RLT buffer (RNeasy Plant Mini Kit) supplemented with 4.5 μL β‐mercaptoethanol, mixed with glass beads and lysed by mechanical disruption using a vortex‐Genie2 with a TurboMix Attachment (2 cycles of 2 min). The lysates were centrifuged (15 s, 4°C, 15,000× g) and processed with the RNeasy Plant Mini Kit (QIAGEN, #74904). To remove genomic DNA contamination, RNA samples were incubated with 4 U of Turbo DNAse (AM1907, Invitrogen) for 1 h at 37°C and then purified and concentrated using the RNeasy MinElute Cleanup Kit (QIAGEN, #74204). The integrity of the isolated RNA was assessed by agarose gel electrophoresis with the Bioanalyzer 2100 system (Agilent Technologies).

The RNA‐seq libraries were prepared using the Illumina Total RNA Prep with Ribo‐Zero Plus kit (Illumina), following the manufacturer's protocol. Library quality control included assessment of fragment size distribution and integrity with the Bioanalyzer 2100, and quantification of DNA concentration using the Qubit DNA HS Assay Kit (Thermo Fisher Scientific). The resulting DNA libraries were sequenced on an Illumina NovaSeq 6000 SP‐200 platform using 2 × 75 bp paired‐end reads. The sequencing was performed by the Genomic Service of CABIMER (Seville, Spain). A total of 121 million reads were obtained, with > 94.8% of bases achieving a quality score of Q30 or higher.

The sequencing reads were aligned to the *Synechocystis* sp. PCC 6803 reference genome (NCBI Assembly: GCF_000009725.1; RefSeq: NC_000911.1) using BWA‐mem2 alignment algorithm from BWA v2.2.1 (Li 2013). Gene‐level raw read counts were generated using the HTSeq‐count function from the HTSeq framework v0.11.0 (Anders et al. 2015). The differential gene expression analysis was performed using the DESeq2 package from Bioconductor (Love et al. 2014) in the R environment. Genes with an adjusted *p* < 0.05 were considered significantly differentially expressed. Genes were functionally annotated using the CyanoGenes webserver (www.cyanogenes.ciccartuja.es).

### 2.7 Oxygen Evolution

Oxygen evolution was measured with a Clark‐type oxygen electrode (Hansatech) at 30°C. Two milliliters of 5 μg chlorophyll *a* (chla) mL^−1^ dark‐adapted cultures were illuminated for 10 min (50 μmol photon m^−2^ s^−1^) with 5 min dark periods before and after light exposure. To prevent carbon limitation, cultures were supplemented with 10 mM of NaHCO_3_ immediately before measurements. When needed, freshly prepared 2,6‐Dichloro‐1,4‐benzoquinone (DCBQ, 0.25 mM) and potassium ferricyanide (2.5 mM) were added to the chamber.

### 2.8 Chlorophyll Fluorescence Analysis and Y(II) Calculations

Chlorophyll *a* fluorescence was measured by pulse‐amplitude‐modulation fluorometry with a Dual‐PAM‐100 (Walz) using 1.5 mL cultures at 5 μg chla mL^−1^ as described in (Ortega‐Martínez et al. 2023). The effective quantum yield of PSII [Y(II)] was determined with the formula (Fm′‐Fs)/(Fm′) where Fm′ is maximal fluorescence and Fs is basal fluorescence, both measured during exposure to actinic light. Other parameters analysed were basal fluorescence in the dark (Fo), qL and qP both relate to the redox state of the plastoquinone pool (lake and puddle models, respectively (Kramer et al. 2004)). These parameters were obtained from the recording of an induction curve, exposing dark‐adapted cultures to actinic light (40 μmol photon m^−2^ s^−1^) followed by a post‐illumination recovery and multiple saturation pulses (5000 μmol photon m^−2^ s^−1^).

### 2.9 NAD(P)H Fluorescence Measurements

The NADPH/9‐AA module of a DUAL‐PAM (Walz) was used to measure light‐induced NADPH redox kinetics on 7.5 μg chla mL^−1^ samples at 30°C by monitoring the changes in fluorescence with excitation at 365 nm and detection between 420 and 580 nm as in (Ortega‐Martínez et al. 2024).

### 2.10 Determination of P700 Redox Kinetics

P700 redox kinetics were measured by pulse‐amplitude‐modulation fluorometry with a Dual‐PAM‐100 (Walz) using intact cells at a concentration of 12.5 μg chla mL^−1^ at room temperature similarly to Shimakawa and Miyake (2018b) and based on the method of Klughammer and Schreiber (2008). Before recording, cells were adapted in the dark for 10 min. During an induction curve with actinic light at 80 μmol photons m^−2^ s^−1^, 300 ms saturation pulses (SP) at 5000 μmol photons m^−2^ s^−1^ with peak emission at 635 nm were supplied at the indicated times to calculate P700 parameters: the PSI quantum yield of photochemical energy conversion [Y(I) = (Pm′‐P)/Pm] and the non‐photochemical energy dissipation due to donor‐side [Y(ND) = P/Pm] and acceptor‐side [Y(NA) = (Pm‐Pm′)/Pm] limitations (Figure S1).

### 2.11 Graphic Representation and Statistical Analysis

In general, visualization of continuous data with the associated standard error of the mean was performed using dplyr and ggplot2 (Tidyverse packages) in R. Figures containing discrete data and statistical analysis were obtained using the software GraphPad Prism 8.0.1. The statistical method used and the number of experimental replicates are described in the figure legends.

## 3 Results

### 3.1 Effect of Glucose Supplementation in *Synechocystis* Under Nitrogen Deprivation

**FIGURE 1:**
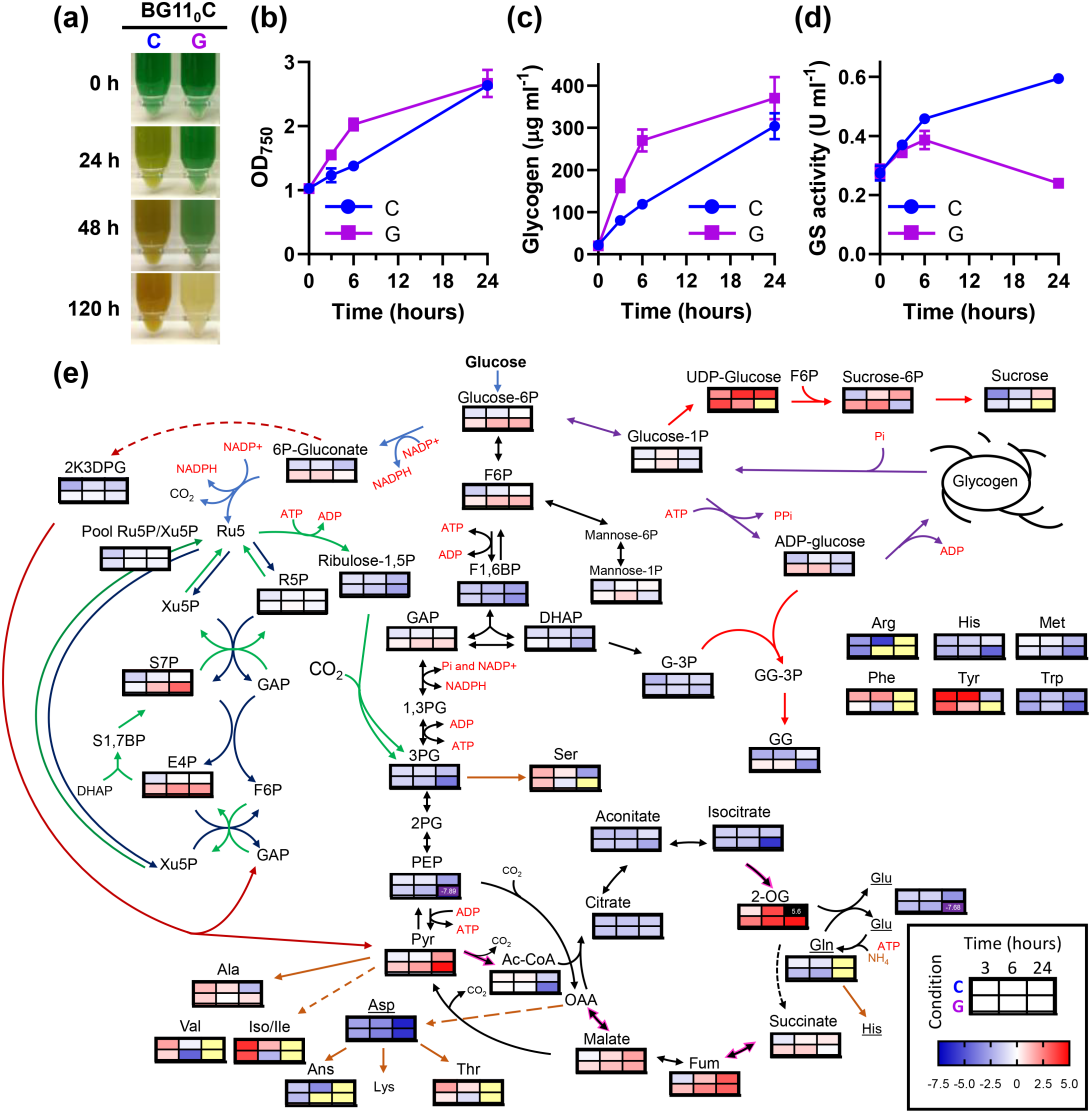
Physiological characterization of the effect of glucose supplementation on the response to nitrogen deprivation in WT *Synechocystis*. WT cells grown photoautotrophically in nitrogen‐replete medium were harvested and resuspended in nitrogen‐free medium (BG11_0_C) at 1 OD_750_. Culture was divided and grown under control (C) conditions or supplemented with 4 mM of glucose (G). (a) Photographs of the cultures at 0, 24, 48 and 120 h after the onset of nitrogen limitation. (b) Growth curves measured as OD_750_. (c) Glycogen content. (d) Glutamine synthetase activity expressed as U mL^−1^. (e) Relative abundance of metabolites measured by LC/MS. Individual heatmaps for each metabolite are embedded within a metabolic network diagram representing the log2 fold‐change of the mean abundance relative to 0 h and normalized to OD7_50_. Metabolites coloured: Not detected (yellow), log2 fold‐change > 5 (black), log2 fold‐change < 7.5 (purple). Measurements in (b–e) were performed at 0, 3, 6 and 24 h after the onset of nitrogen limitation. Data in (b–e) represent the mean ± SEM of 6 and 4 independent biological replicates, respectively. 2K3DPG, 2‐keto‐3‐deoxy‐phosphogluconate; 2‐OG, 2‐oxoglutarate; 3PG, 3P‐glycerate; AcCoA, acetyl‐CoA; DHAP, dihydroxyacetone‐P; E4P, erythrose‐4P; F1,6BP, fructose‐1,6‐BP; F6P, fructose‐6P; Fum, fumarate; G‐3p, glycerol‐ 3P; GAP, glyceraldehyde‐3P; GG, glucosylglycerol‐3P; OAA, oxalacetate; PEP, phosphoenolpyruvate; Pyr, pyruvate; R5P, ribose‐5P; Ru‐5P/Xu‐5P, ribulose‐5P/xylulose‐5P pool; S7P, sedoheptulose‐7P.

To address the mechanism by which glucose inhibits the chlorosis process, we analyzed the physiological response of *Synechocystis* to nitrogen deprivation in the presence of glucose. WT cells, previously grown photoautotrophically in nitrogen‐replete medium, were transferred to nitrogen‐depleted medium in the absence (C, control) or presence of 4 mM glucose (G, glucose). As expected, while the control culture started chlorosis in response to nitrogen deprivation, glucose supplementation completely blocked bleaching, and the cells maintained their pigmentation until 48 h (Figure 1a), in agreement with previous results (Elmorjani and Herdman 1987).

Glucose supplementation induced rapid physiological alterations in the response to nitrogen deprivation that ultimately compromised viability. Within the first 6 h of nitrogen deprivation, we observed a rapid rise in OD_750_ (OD_750_ from 1 to 2.1 ± 0.25) and glycogen reserves (269 μg mL^−1^); representing a 2.28 fold‐change increase in glycogen content compared to the control condition (118 μg mL^−1^) (Figure 1b,c). This was accompanied by a progressive consumption of glucose from the medium, with approximately 1.2 ± 0.18 mM left in the medium after 24 h (Figure S2a).

However, despite this physiological response and maintenance of the phycobiliprotein peak (625 nm), the whole‐cell absorption spectra presented a flattening pattern (Figure S2b) indicative of cell stress and compromised cellular viability. This was further corroborated by the significant increase in reactive oxygen species (ROS) after 24 h (Figure S2c), and by the whitening of the cultures after 120 h of incubation (Figure 1a).

Despite the lack of bleaching, glucose‐supplemented cells sensed the nitrogen depletion and responded accordingly, as indicated by two key markers of nitrogen deficiency: increased glutamine synthetase (GS) activity and PII hyperphosphorylation (Figures 1d and S3). These two responses occurred similarly during the first hours of nitrogen deprivation both in the presence and absence of glucose. Ultimately, after 24 h in the presence of glucose, GS activity was decreased, most likely due to the lethality of this condition, with general protein loss and ROS increase (Figures S2b,c and S3) (Robles‐Rengel et al. 2019).

Metabolomic analysis confirmed the expected increase in the nitrogen status sensor 2‐OG, key in the responses of GS activity and PII phosphorylation state, under nitrogen‐deprived conditions both in the presence and absence of glucose (Figure 1e; Table S1). However, important alterations were observed in the metabolome of glucose‐supplemented cells. In contrast to control nitrogen‐deprived cells, glucose supplementation led to a progressive accumulation of glycolytic and pentose phosphate pathway intermediates, including glucose‐6‐phosphate (G6P), fructose‐6‐phosphate (F6P), sedoheptulose‐7‐phosphate (S7P), erythrose‐4‐phosphate (E4P), and pyruvate, as well as the glycogen precursor ADP‐glucose (Figure 1e; Table S1).

In the absence of glucose, nitrogen starvation resulted in a transitory increase in some amino acids and a severe depletion of all the analysed pools of amino acids after six to 24 h (Figure 1e; Table S1). This depletion occurred earlier in the presence of glucose, with minimal levels of glutamine, threonine, valine, phenylalanine or arginine detected after the first 3 h. Notably, methionine, an essential initiator of protein synthesis, was nearly undetectable after 24 h (Figure 1e; Table S1).

**FIGURE 2:**
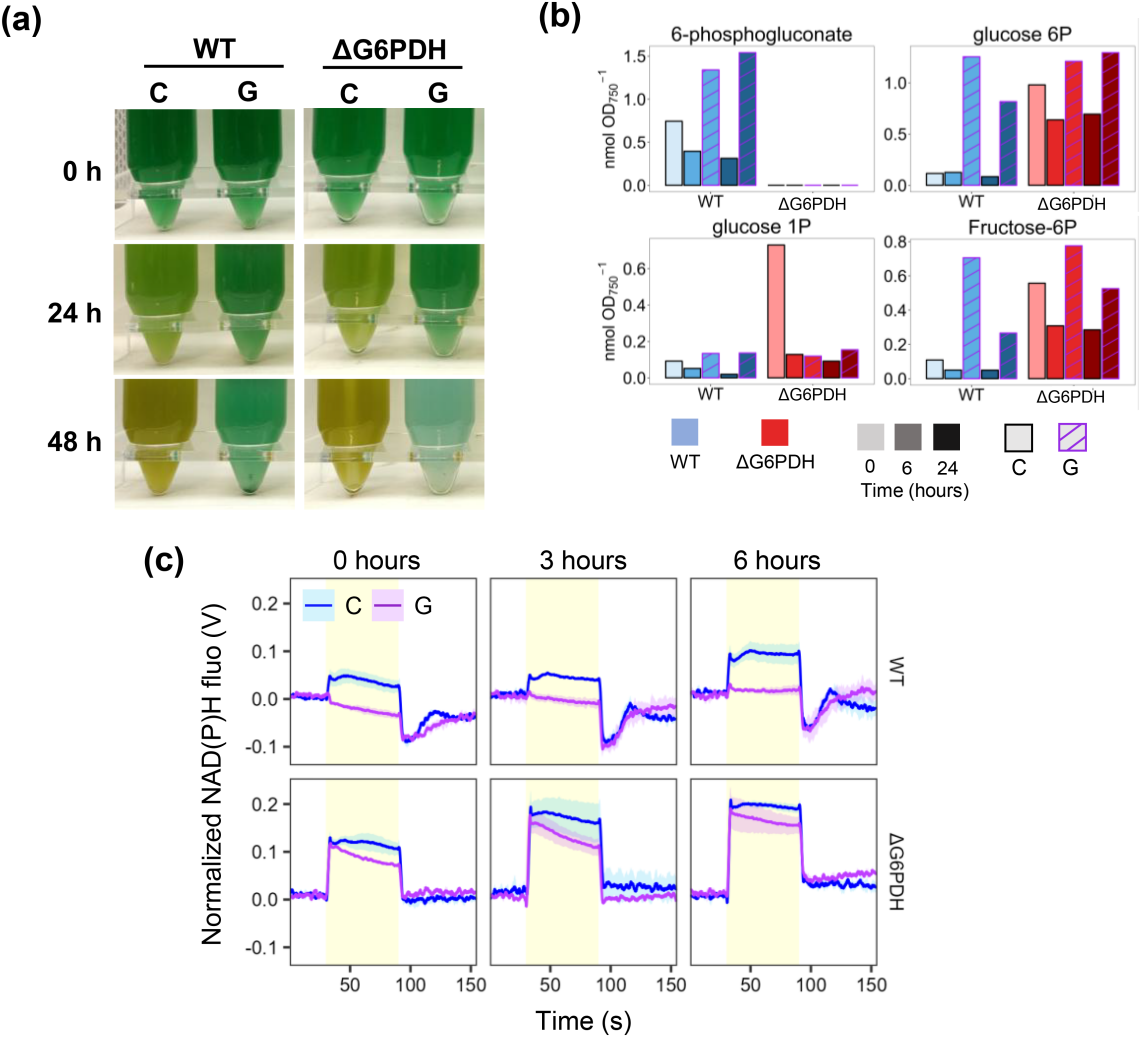
Effect of glucose supplementation on the response to nitrogen deprivation of the ΔG6PDH mutant, lacking gluocose‐6P dehydrogenase. WT and ΔG6PDH strains grown in nitrogen‐replete medium were harvested and resuspended in nitrogen‐free (BG11_0_C) medium at 1 OD_750_. Cultures were divided and grown under control (C) conditions or supplemented with 4 mM of glucose (G). (a) Photographs of the cultures at 0, 24 and 48 h after the onset of the experiment. (b) Measurements of metabolites from the first steps in glucose assimilation by LC/MS at 0, 6 and 24 h. (c) NAD(P)H fluorescence light‐dependent kinetics of WT and ΔG6PDH cultures (12.5 μg chl mL^−1^) at 0, 3 and 6 h after the onset of the experiment. Data in (b) are from one experiment; data in (c) represent the mean ± SEM of 3 independent biological replicates.

Interestingly, the observed increase in G6P and 6PG, along with the subsequent generation of reducing power (NADPH) through the oxidative pentose phosphate (OPP) shunt, was proposed as a potential key factor in preventing chlorosis (Gründel et al. 2012). To test this hypothesis, we disrupted the OPP shunt by generating a mutant strain lacking glucose‐6‐phosphate dehydrogenase (G6PDH). The WT and the ΔG6PDH mutant strain were subjected to nitrogen depletion under control or glucose supplementation conditions (Figure 2). Like the WT, the ΔG6PDH strain could not undergo bleaching when supplemented with glucose, losing the chlorophyll pigments within 48 h (Figure 2a) despite exhibiting undetectable 6PG levels (Figure 2b). In fact, while the WT maintained the typical NAD(P)H light‐dependent synthesis trace even 24 h after nitrogen depletion, the presence of glucose caused an immediate change in the NAD(P)H kinetics with no increase in its associated fluorescence (Figure 2c), resembling the similar effect observed in the control (Ortega‐Martínez et al. 2024). However, as expected due to the impediment of NADPH synthesis by the OPP shunt, the ΔG6PDH strain presented a light‐dependent increase in the NAD(P)H fluorescence kinetics in the presence of glucose, although the trace is slightly affected compared to the control condition (Figure 2c). This suggests that 6PG accumulation and potential overreduction of the NADPH pool are not the cause behind the lack of bleaching. Interestingly, while disrupting the OPP shunt resulted in mild alterations in metabolites related to the glucose‐6‐P crossroads, it did not impact the bleaching process (Figure 2b). These effects of the ΔG6PDH on the NAD(P)H fluorescence kinetics and metabolic alterations were also observed in previous studies (Maruyama et al. 2019; Hatano et al. 2022).

Overall, these results indicate that glucose intake in nitrogen‐deprived medium fuels glycogen reserves. However, achieving high glycogen levels early during nitrogen depletion might exceed its total storage capacity, leading to a metabolic overflow like what is observed when glycogen synthesis is limited and thus causing cell death.

### 3.2 Comparative Transcriptome Changes Induced by Glucose Upon Nitrogen Depletion

As the phosphorylation state of PII indicated that glucose did not alter nitrogen sensing, we next proceed to analyze its effect on the transcriptomic response by RNA‐seq. *Synechocystis* grown photoautotrophically in nitrogen‐replete medium (0 h) were transferred to nitrogen‐depleted media, either without (C, control) or with 4 mM glucose (G, glucose), and sampled after 3 and 6 h. A total of 3228 transcripts were detected (Table S2). At the 3‐h time point, we observed a comparable number of differentially expressed genes (DEGs; fold change > 1.5, adjusted *p* < 0.05) in both conditions, with 428 DEGs in Control and 575 DEGs in Glucose compared with nitrogen‐repleted sample (0 h) (Table S3). By 6 h under nitrogen deprivation, the transcriptional responses began to diverge more markedly, with 480 DEGs in the control condition and 921 DEGs in the glucose‐supplemented condition.

**FIGURE 3:**
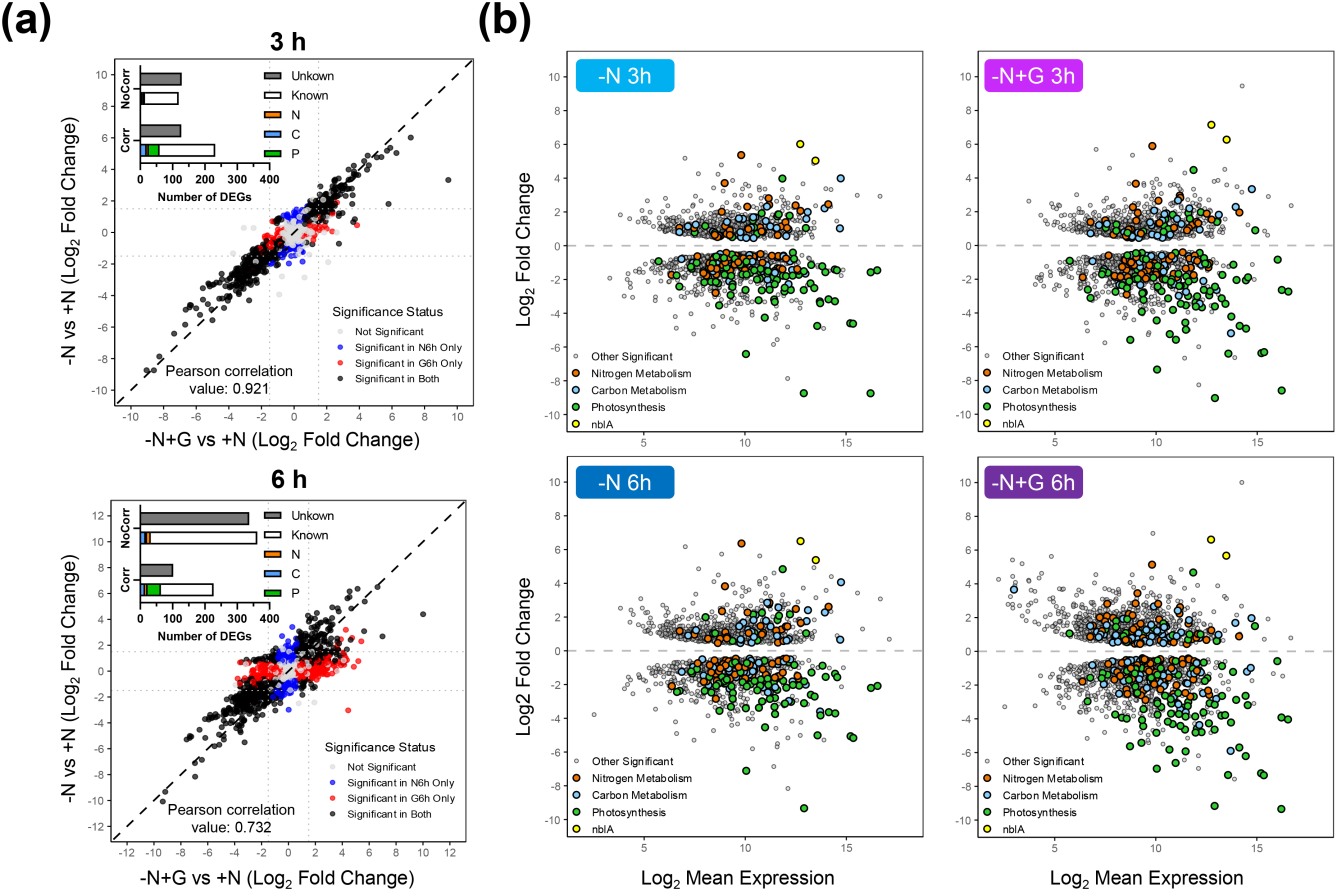
Effect of glucose supplementation on the transcriptomic response of *Synechocystis* to nitrogen deprivation. WT cells grown photoautotrophically in nitrogen‐replete medium (0 h) were harvested and resuspended in nitrogen‐free medium (BG11_0_C) at 1 OD_750_. Culture was divided and grown under control (C) conditions or supplemented with 4 mM of glucose (G). (a) Scatter plots showing the correlation of gene expression between C and G conditions after 3 and 6 h of nitrogen deprivation. Pearson correlation coefficients are indicated at the bottom of each plot. Functional annotation of known and unknown differentially expressed genes (DEGs) that showed correlation (Corr) or no correlation (NoCorr) is summarized in the top‐left insets. Among the annotated genes, those involved in carbon metabolism (Carbon), photosynthesis and respiration (Photo), and nitrogen metabolism (N) are highlighted in blue, green, and orange, respectively. (b) MA plots showing log_2_ fold‐change versus mean expression for each gene under nitrogen deprivation in control (C) conditions or supplemented with glucose (G) after 3 and 6 h of treatment, compared to initial nitrogen‐replete state (0 h). The contrasts shown are: C 3 h versus 0 h, C 6 h versus 0 h, G 3 h versus 0 h, and G 6 h versus 0 h. All genes represented (dots) show significant differential expression (adjusted *p* < 0.05). Genes annotated with functional categories related to nitrogen deprivation response are highlighted as coloured dots: Carbon metabolism (blue), photosynthesis and respiration (green), nitrogen metabolism and assimilation (orange), and *nblA* genes (yellow). The remaining significant genes are shown in grey.

Despite these differences, the global transcriptomic responses to nitrogen starvation in the presence and absence of glucose remained highly correlated across all genes, particularly at 3 h (Pearson correlation coefficient: 0.92) and to a lesser extent at 6 h (Pearson correlation coefficient: 0.79) (Figure 3a). When focusing specifically on genes associated with the chlorosis program (230 genes related to photosynthesis and respiration, carbohydrate and carbon central metabolism and nitrogen assimilation and metabolism; Table S4), we observed that these genes consistently exhibited similar patterns of differential expression in both treatments (Figures 3b and S1). This indicates that the observed transcriptomic divergence is largely driven by other genes not directly linked, according to current literature, to chlorosis‐related cellular processes. Notably, this divergence was more pronounced at 6 h, where a substantial proportion of genes showing differential expression patterns between conditions were annotated as hypothetical or of unknown function (NC in Figure 3a), suggesting that glucose availability may importantly affect the expression of poorly characterized regulatory or metabolic pathways.

The well characterised transcriptional response to nitrogen deprivation (Krasikov et al. 2012; Matthias et al. 2014; Carrieri et al. 2017; Giner‐Lamia et al. 2017; Esteves‐Ferreira et al. 2018) occurred regardless of glucose presence. This common response includes (Figure 3): (i) Upregulation of nitrogen uptake systems, including those for ammonium (*amt1‐3; sll1017, sll0537 and sll0108*), urea (*urtA‐E*; *slr0447, slr1200, slr1201, sll0764 and sll0374*), and nitrate (*nrtD, sll1082*). (ii) Adjustment of nitrogen metabolism, with upregulation of *glnN* (*slr0288*), encoding GSIII, and downregulation of genes encoding GS inactivating factors IF7 (*gifA, ssl1911*) and IF17 (*gifB, sll1515*), in agreement with GS activity measurements (Figure 1d). This also included repression of the genes encoding the ferredoxin‐nitrite reductase (*nirA, slr0898*), and the PII protein interactor A (*pirA, ssr0692*), mediating arginine synthesis. (iii) Coordination with carbon metabolism, with strong upregulation of *cfrA/pirC* (*sll0944*), a nitrogen‐responsive metabolic regulator controlling carbon flow (Muro‐Pastor et al. 2020). Other transcriptional changes related to the regulation of carbon metabolism were downregulation of genes coding for ADP‐glucose pyrophosphorylase (*agp*, *slr1176*), phosphoribulokinase (*prk*, *sll1525*), and both RuBisCO subunits (*rbcS, slr0012* and *rbcL, slr0009*). (iv) Repression of photosynthesis‐related genes, including genes coding for phycobiliproteins such as allophycocyanin (APC genes) and phycocyanin (cpcA‐D, G) chains, subunits or linkers, photosystems (psa and psb subunits), several ATP synthase subunits (atpA‐I), or proteins of the carbon dioxide concentrating mechanism (CCM) (*sll1029*, *sll1030*). In contrast, genes encoding subunits of NADH dehydrogenase (*slr0851*), and cytochrome oxidases (*cyd*, *slr1380*; *slr1379*; *cta, slr1136*; *sll1899*) were upregulated. (v) Finally, of special interest is the strong upregulation of *nblA* genes (*ssl0452* and *ssl0453*), required for phycobiliproteins degradation. These were among the most upregulated genes (log_2_ FC > 5) in both conditions and times tested, indicating that the transcriptional response of phycobiliprotein degradation was promoted (Table S4; Figure S4). It is also important to note that many of these genes belong to the NtcA regulon (Giner‐Lamia et al. 2017). Therefore, these data confirm that glucose treatment does not interfere in the transcriptomic response to nitrogen deprivation, and specifically with the NtcA‐mediated response.

However, glucose supplementation also led to differential transcript changes (Table S4; Figure S4). After 3 h, only 46 genes presented statistically significant differences with most of them being of unknown function. However, the two genes most highly induced by glucose were *fabG* (*sll0330*), implicated in fatty acid biosynthesis and considered a potential PhaB (acetoacetyl‐CoA reductase; Zhang et al. 2017), and the PGR5‐like homolog *ssr2016*. These two genes were also upregulated in the absence of glucose, but to a lesser extent. Another DEG in glucose is the Site‐2‐Protease *sll0528*, related to carbon/nitrogen homeostasis and stress response (Lei et al. 2014; Lin et al. 2025), being highly expressed compared with the control condition (log_2_ FC of 2.3 and 3.91 after 3 and 6 h). In addition, several heat shock (*hsp17, sll1514* and *htpG*, *sll0430*) and high‐light‐inducible genes (*ssr2595* and *ssl2542*) were among the most notorious changes detected. Conversely, several genes related to the CCM and PBS assembly were specially repressed in the presence of glucose. Some central carbon metabolism genes such as those encoding pentose‐5‐phosphate‐3‐epimerase (*sll0807*), phosphoglucomutase (*sll0726*), phosphoglycerate mutase (*slr1124*) and acetyl‐coenzyme A synthetase (*sll0542*) presented a specifically depleted expression with glucose. After 6 h, an additional set of genes was also induced, mainly with unknown function or related to stress response.

### 3.3 Photosynthetic Characterization During Nitrogen Deprivation and Glucose Supplementation

**FIGURE 4:**
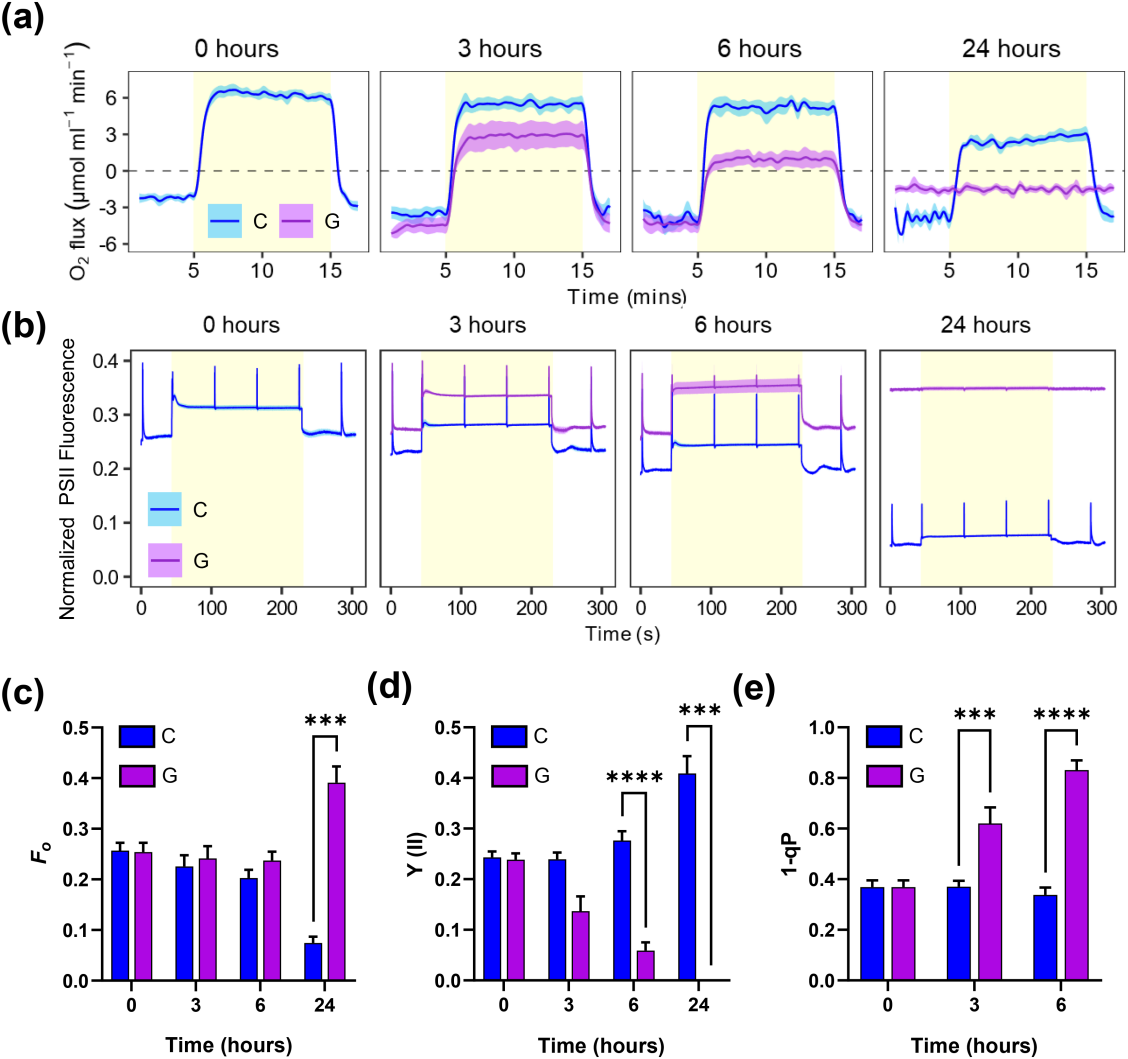
PSII characterization during the response to nitrogen deprivation with glucose supplementation in WT *Synechocystis*. WT cells grown in nitrogen‐replete medium (BG11C) were harvested and resuspended in nitrogen‐free medium (BG11_0_C) at 1 OD_750_. The culture was divided and grown under control (C) conditions or supplemented with 4 mM of glucose (G). (a) Oxygen evolution rates of cultures (5 μg chl mL^−1^) at 0, 3, 6 and 24 h time points after nitrogen deprivation illuminated with 50 μmol photons m^−2^ s^−1^. (b) Chlorophyll fluorescence induction curves of C and G cultures at timepoints 0, 3, 6 and 24 h after nitrogen deprivation. Fluorescence values were normalized to the minimum value of fluorescence trace. Parameters obtained from the traces of chlorophyll fluorescence induction curves are summarized in (c) basal fluorescence in the dark (*F*_*o*_), (d) effective quantum yield of PSII Y(II), and (e) 1‐qP estimations. Data represent the mean ± SEM of 5 independent biological replicates.

**FIGURE 5:**
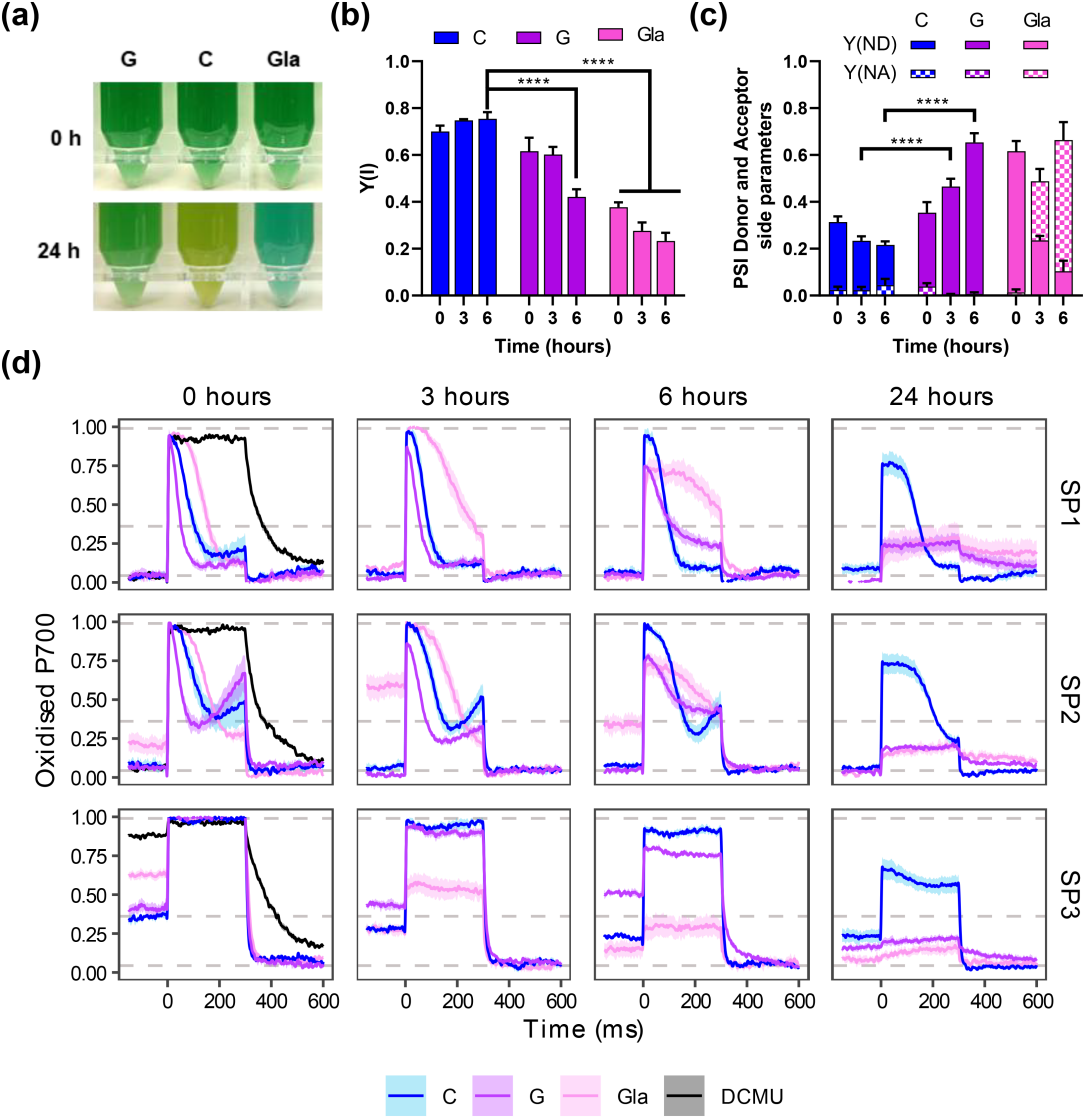
P700 characterization during the response to nitrogen deprivation with glucose and glycolaldehyde supplementation in WT *Synechocystis*. WT cells grown in nitrogen‐replete medium (BG11C) were harvested and resuspended in nitrogen‐free medium (BG11_0_C) at 1 OD_750_. The culture was divided and grown under control (C) conditions or supplemented with 4 mM of glucose (G) or 5 mM of glycolaldehyde (Gla). (a) Photographs of the cultures at 0 and 24 h. (b) Quantification of PSI effective quantum yield [Y(I)] during the SP3 of the induction curve for all the conditions at 0, 3 and 6 h after nitrogen depletion. (c) Quantification of PSI donor‐side limitation [Y(ND)], and acceptor‐side limitation [Y(NA)] during the SP3 of the induction curve for all the conditions at 0, 3 and 6 h after nitrogen depletion. (d) Analysis of the redox state of P700 by the changes in near‐infrared absorbance upon the saturation pulses (SP1‐3; see Figure S1) during an induction curve in a Dual‐PAM‐100 for all the conditions at 0, 3, 6 and 24 h after nitrogen depletion. For PSII inhibition, 20 μM DCMU was added to control culture at 0 h. Dashed lines represent different P700 oxidized states of the control at time 0 h. From bottom to top: Basal oxidation level, the steady‐state P700 oxidation level under actinic light (P) and maximum P700 oxidation level (Pm´) under a saturation pulse during actinic light. Each trace was baselined by subtracting the smallest value within the trace and normalized to the Pm (maximum value of the SP2) of each condition independently at time 0 h. Data represent the mean ± SEM of 4 independent biological replicates. Statistical significance was denoted ****p < 0.0001; otherwise indicates no significance (two‐way ANOVA).

Prior research has demonstrated that metabolic overflow (specifically because of glycogen synthesis deficiency) impairs photosynthesis (Holland et al. 2016; Ortega‐Martínez et al. 2023), which can block the bleaching process (Yoshihara and Kobayashi 2022). To evaluate the impact of glucose on photosynthetic performance during nitrogen deprivation, we analysed oxygen evolution in cultures transferred to nitrogen‐depleted medium in the absence (C, control) or presence of 4 mM glucose (*G, glucose*) (Figures 4 and 5). In the control, the O_2_ evolution of the cultures retained 80% ± 9% and 40% ± 5% of the initial rate by six and 24 h, respectively (Figure 4a). In contrast, glucose supplementation decreased O_2_ evolution, reaching 15% ± 10% of the initial rate by 6 h and becoming abolished by 24 h. However, this impairment was alleviated by adding the artificial PSII electron acceptor DCBQ, indicating that the oxygen‐evolving complex of PSII remained functional after 6 h of nitrogen deprivation in glucose‐supplemented cultures (Figure S5).

Chlorophyll fluorescence analysis revealed a progressive decrease in dark‐adapted basal fluorescence (*F*_o_) during nitrogen depletion for the control condition under photoautotrophic conditions (Figure 4b). This decrease is likely due to the degradation of the PBS (Figure S2b), whose phycocyanin contributes to the basal fluorescence (Ogawa et al. 2017). The effective quantum yield of PSII [Y(II)] under moderate actinic light remained stable during the first 6 h of nitrogen depletion, consistent with the O_2_ evolution (Figure 4a), but increased to ~0.4 by 24 h (Figure 4b). This rise is also likely due to PBS degradation (Figure S2b), because of the aforementioned effect on *F*_o_ and a decrease in excitation energy transfer to PSII (Ogawa and Sonoike 2016). In contrast, glucose treatment increased Fs but not Fm′, without a detectable decrease in *F*_o_ (Figure 4c). Consequently, there was a severe decrease in Y(II), reaching values as low as 0.05 ± 0.016 after 6 h, which is 18% of the value of the control condition at that time point (Figure 4d). After 24 h, cells displayed no response to actinic light or saturating pulses, consistent with protein degradation and cell death.

Analysis of the coefficient of photochemical quenching based on the puddle model (qP) of antennae connectivity revealed progressive PSII reaction centre closure and increased Q_A_ reduction with glucose, suggesting an increasingly reduced plastoquinone (PQ) pool (Figure 4e). Similar trends were observed using the lake model estimation (1‐qL) (Figure S6). These parameters are affected by phycocyanin fluorescence levels (Ogawa and Sonoike 2016); however, PBS had not decreased enough at 6 h to account for these differences and an unchanging 1‐qP parameter during 6 h of nitrogen depletion under control condition, where there was not PBS degradation, indicated an unchanged redox state of the PQ pool.

We also measured Q_A_^−^ re‐oxidation kinetics after dark adaptation and a single turnover saturating flash, and these results were consistent with our consideration of the qP parameter. Upon glucose treatment, the fluorescence decay slowed compared to the control, and at 6 h of treatment the QA^−^ re‐oxidation kinetics resembled the effects of the photosynthesis inhibitor DCMU (Figure S7).

To elucidate the impact of this altered photosynthetic capacity on PSI performance, we evaluated P700 redox kinetics with light induction curves in a Dual‐PAM‐100 (Figure 5). As a control for PSI acceptor side limitation, we included treatment with 5 mM glycolaldehyde (Gla), an inhibitor of phosphoribulokinase that arrests the CCB cycle by preventing RuBisCO substrate regeneration. Similar to other treatments that interfere with photosynthesis, Gla treatment prevented the bleaching process causing the culture to turn blue after 24 h of nitrogen depletion (Figure 5a). The NAD(P)H kinetics showed a constant increase in NAD(P)H fluorescence during the light period (Figure S8) instead of achieving a steady state, as observed in other conditions (Figure 2), which confirms the effectiveness of the treatment.

To evaluate the redox state of PSI, we analysed the effect of saturating pulses in the dark (SP1), after far‐red illumination (SP2) and during actinic illumination (SP3) (Figure S1). Under control conditions at the onset of nitrogen depletion, P700 presented typical redox kinetics during the SPs (Figure 5d). In the dark, the SP1 caused a fast oxidation of the fully reduced P700 reaction center to its maximal oxidized state, which then was reduced by electrons from PSII (reduction phase absent in the SP1 and SP2 P700 redox kinetics of DCMU‐treated cells). This reduction persisted during the rest of the SP due to limitations on the P700 acceptor side primarily due to the lack of actinic light to switch on the CBB cycle (Shimakawa and Miyake 2018a). Under far‐red illumination (SP2), which preferentially excites PSI, the P700 redox kinetics also featured a re‐oxidation phase after 150 ms by the action of the flavodiiron proteins (FLVs) (Shimakawa and Miyake 2018b). However, under actinic light, the P700 pool was partially oxidized and became fully oxidized during SP3, due to lack of limitation on the acceptor side, just to return to a reduced state after the SP (Shimakawa and Miyake 2018a). Addition of DCMU caused a delay in the reduction of P700 after the SPs and an almost complete oxidized state during actinic light (SP3) due to the restricted electron donation from PSII (Figure 5d).

Supplementation of the nitrogen‐deprived cultures with glucose or Gla had immediate effects on the redox kinetics of P700. Glucose quickened the reduction of P700 during SP1 and SP2, and there was a stronger re‐oxidation associated with the FLVs activities during SP2. Conversely, Gla treatment slowed the reduction of P700 during SP1 and SP2, and had a partially oxidized P700 pool during the actinic light prior to SP3. Notably, Gla treatment also increased the steady‐state oxidation level of P700 during far‐red light (SP2), especially after 3 h of incubation, suggesting that Gla not only inhibits the CBB cycle but also interferes with the balance between linear and cyclic electron transport, as previously suggested (Kusama et al. 2022).

Through 3 and 6 h of nitrogen deprivation, control cells maintained their P700 redox kinetics with a progressive decrease in the level of oxidized P700 (Figure 5d). Glucose supplementation progressively increased the oxidized pool of P700 during actinic light (SP3), decreased the maximum oxidizable P700 pool, decreased Y(I) (Figure 5b), and increased limitation on the P700 donor side [Y(ND)] (Figure 5c). At 24 h of nitrogen deprivation and treatment with glucose or Gla, the P700 photooxidation signal was negligible.

In summary, these data suggest a restricted electron flow in the presence of glucose when nitrogen is depleted, which progressively results in the oxidation of P700 and downstream components, as confirmed by a significant decrease in maximum Fd reduction after a multiple‐turnover flash during intense actinic light exposure (Figure S9). Therefore, ensuring a threshold of the P700 pool in a reduced state during illumination and maintaining the redox poise on the last components of the PETC seem to be critical for performing bleaching.

### 3.4 Effect of Glucose Addition at Different Times During the Bleaching Process

**FIGURE 6:**
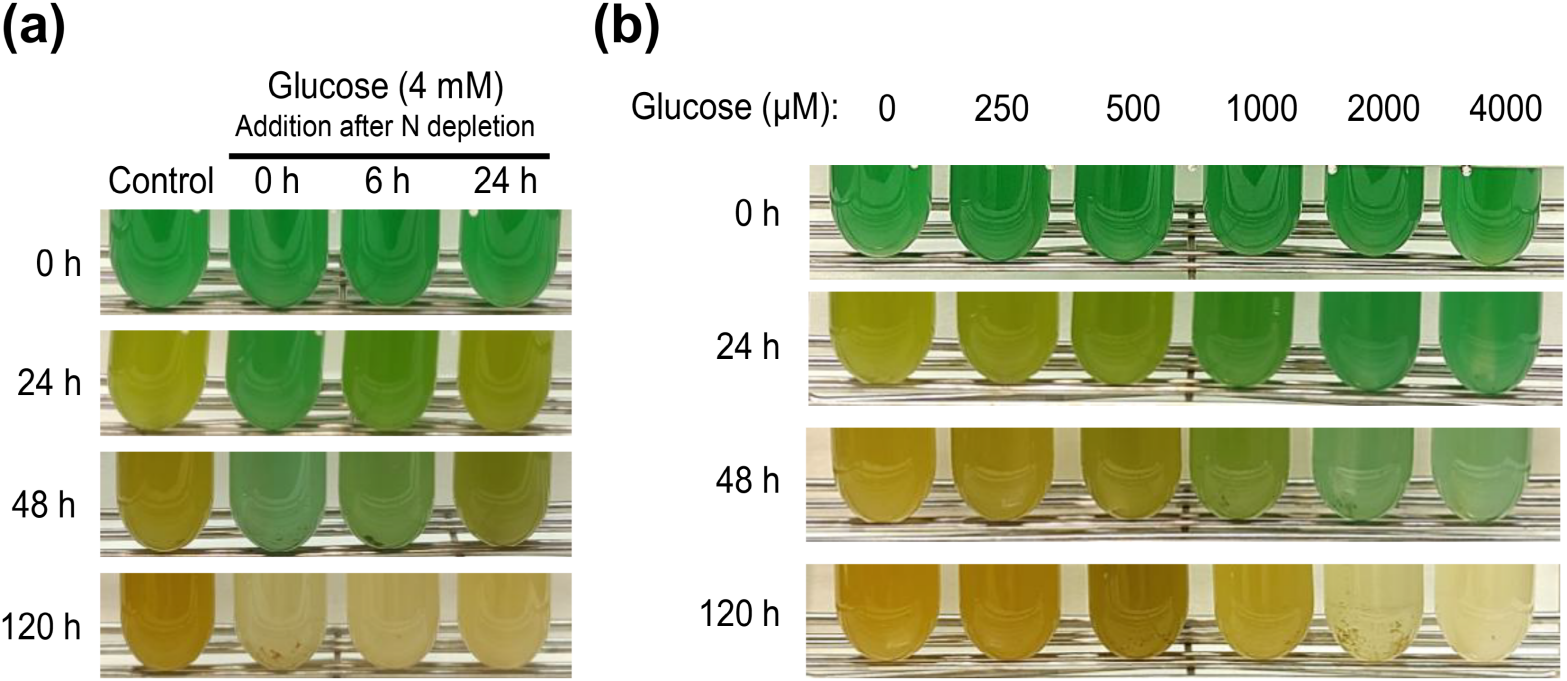
Effect of glucose concentration and time of addition on the bleaching process in response to nitrogen deprivation in WT *Synechocystis*. (a) Photographs of the growth of the WT strain cultured in nitrogen‐deplete medium (BG11_0_C) with addition of glucose at certain times after nitrogen deprivation. (b) Photographs of the growth of the WT strain cultured in BG11_0_C with different amounts of glucose added at the onset of nitrogen deprivation, ranging from 250 to 4000 μM.

To determine whether the inhibitory effect of glucose on the bleaching process is restricted to its onset or if it can halt the process after it has been initiated, glucose was added to WT cultures at different times or concentrations after nitrogen removal. Nitrogen depletion response was halted when glucose was added regardless of the stage of the bleaching process, even after phycobiliproteins' degradative process had already started (Figure 6a). This indicates that the inhibitory effect of glucose on the bleaching process is not restricted to the onset of the response to nitrogen deprivation. Interestingly, the response was dose‐dependent and reversible: if the glucose provided was below a certain threshold able to be consumed by the cultures (1 mM glucose), then bleaching proceeded with a proportional time delay (Figure 6b). This indicates that cells can accommodate to a certain level of disturbance in metabolic intermediates' levels.

## 4 Discussion

In photosynthetic organisms, maintaining balanced carbon pools is crucial to adapt to environmental changes while preventing electron transport dysregulation. *Synechocystis* can perform chlorosis (bleaching) under nitrogen deprivation or can grow photomixotrophically with glucose, both conditions requiring substantial metabolic adaptation and restructuring of the photosynthetic machinery (Ortega‐Martínez et al. 2023, 2024). Glycogen plays a critical role in these processes, acting as a metabolic buffer that coordinates source–sink relationships within the cell. In fact, nitrogen deficiency adaptation is characterized by an increase in the C/N ratio, leading to accumulation of glycogen, partially supported by carbon recycled from degraded phycobiliproteins (Figure 1c and Hasunuma et al. 2013; Ortega‐Martínez et al. 2023). However, glucose supplementation under nitrogen deprivation further enhances the C/N ratio and doubles glycogen reserves within 6 h (Figure 1c), preventing PBS degradation and bleaching (Figure 1a), in agreement with a previous report (Elmorjani and Herdman 1987).

This suggests the combination of external glucose and PBS‐derived carbon may exceed the buffering capacity of glycogen, thereby disrupting the chlorosis program. Consistent with this, we observed substantial alterations in carbon partitioning and accumulation of key metabolic intermediates (e.g., S7P, E4P, F6P, pyruvate, 2‐OG, 6PG, malate, fumarate, succinate; Figure 1e), indicating metabolic overflow. Such imbalances likely interfere with allosteric regulations and metabolic checkpoints essential for the progression of chlorosis as a nitrogen depletion response. This conclusion is supported by the non‐bleaching phenotypes of mutants in enzymes such as alanine dehydrogenase or AGP, which also generate metabolic alterations (Lahmi et al. 2006; Carrieri et al. 2012; Gründel et al. 2012; Hickman et al. 2013; Ortega‐Martínez et al. 2023).

Despite the lack of bleaching and the presence of a metabolic imbalance, the transcriptomic response to nitrogen deprivation in the presence of glucose was remarkably similar to that observed under nitrogen‐depleted conditions (Figure 3). This response was consistent with earlier studies in *Synechocystis* during chlorosis (Krasikov et al. 2012; Matthias et al. 2014; Carrieri et al. 2017; Giner‐Lamia et al. 2017; Esteves‐Ferreira et al. 2018), where nitrogen deprivation induced genes involved in nitrogen uptake and transport systems, as well as *nblA* expression, while repressing transcripts for phycobiliproteins and carbon fixation pathways. This suggests that post‐transcriptional or metabolic‐level regulation, rather than transcriptional regulation alone, contributes to the suppression of bleaching by glucose, independently of *nblA* expression. This conclusion is supported by non‐bleaching mutants, such as the AGP (*glgC*) glycogen synthesis mutant (Hickman et al. 2013; Carrieri et al. 2017) or the mutant lacking the GntR‐family regulator *sll1961* (Ozaki et al. 2007; Sato et al. 2008), expressing *nblA* during nitrogen deprivation but not bleaching. The observation that glucose addition halts bleaching even after the process has been initiated (Figure 6) supports the existence of a superimposed mechanism of chlorosis control independent of the sensing and transcriptional response to nitrogen deficiency. Strikingly, the combined stress of nitrogen depletion and glucose supplementation in wild‐type cells mirrors the behaviour of glycogen‐deficient mutants (ΔAGP, ΔPGM) under these stresses separately, showing impaired viability, metabolic overflow and decreased photosynthetic activity (Ortega‐Martínez et al. 2023, 2024). This suggests that the chlorosis program might be directly influenced and disturbed by alterations in these processes, alongside the established role of the NtcA transcription factor, nitrogen sensing and C/N ratio. Supporting this, our photosynthetic measurements of the nitrogen‐depleted WT culture supplemented with glucose showed a rapid decline in performance upon glucose addition during nitrogen deprivation (Figure 4), similar to observations in glycogen mutants under nitrogen deficiency or glucose supplementation (Carrieri et al. 2012; Ortega‐Martínez et al. 2023, 2024). Both oxygen evolution and Y(II) were already affected after 3 h of glucose treatment, indicating that most PSII reaction centers are closed (Figure 4a–d) and Q_A_ reduced (Figures 4e, S6 and S7), with an expected reduced PQ pool. This photosynthetic condition agrees with the interpretation that nitrogen deficit acclimation requires a fully functional photosynthetic apparatus driven by actinic light, since PSII inhibition by DCMU or dark cultivation impedes the bleaching process (Salomon et al. 2013; Yoshihara and Kobayashi 2022).

An over‐reduced PQ pool could disrupt electron flow, prior to Cyt *b*_6_ *f*, via the RISE (reduction‐induced suppression of electron flow) mechanism (Shimakawa et al. 2018), due to a lack of free oxidized PQ available to participate in the Q cycle at Cyt *b*_6_ *f*. However, overall data from different sources indicate that the PQ pool is not governing the bleaching process. Firstly, DCMU and DBMIB, which have opposite effects on the redox state of the PQ pool, causing oxidation (DCMU) or reduction (DBMIB), have similar effects in preventing bleaching (Yoshihara and Kobayashi 2022). Secondly, the similarities from nitrogen‐deplete conditions supplemented with glucose and previous data of photomixotrophic conditions in glycogen synthesis ΔAGP and ΔPGM mutants, which presented no stalling in the Q cycle (Ortega‐Martínez et al. 2024), suggest that it is unlikely the PQ pool is maximally reduced. Thirdly, disrupting the OPP shunt by deleting the G6PDH enzyme (ΔG6PDH strain), and thus limiting the NADPH synthesis to the FNR reaction, did not ameliorate the impairments in the bleaching process caused by the glucose supplementation (Figure 2). Fourthly, we cannot confirm the redox state of the PQ pool through our measurements here since estimations through qL and qP parameters (Figures 4e and S6) are indirect; rather, they infer it from fluorescence parameters related to Q_A_ whose re‐oxidation is slowed with glucose (Figure S7) and is a known limitation of fluorescence‐based proxies (Khorobrykh et al. 2020). This aligns with DCMU‐treated cells, where Q_A_ is reduced but the PQ pool remains mostly oxidized due to the blocked Q_B_ site within PSI, while fluorescence parameters qP and qL would indicate a reduction of the PQ pool.

Further insight came from P700 and Fd redox measurements. Glucose‐supplemented, nitrogen‐depleted cultures showed a decline in Y(I), increased donor‐side limitation [Y(ND)], and P700^+^ accumulation during actinic illumination (Figure 5). This aligns with decreased electron flow from PSII to PSI and suggests that a redox signal provided by a sufficient electron transfer from PSI may be a prerequisite to proceed with chlorosis, while a diminished P700/P700^+^ ratio could prevent bleaching. Nevertheless, under nitrogen‐depleted conditions, the quantitatively strong photosynthetic electron sink from Fd, which supposes nitrate reduction to ammonia, is not active. This scenario contributes to the reduction of the Fd pool and thus diminishes the oxidation of its electron donor PSI (P700), becoming a key component to trigger the response to nitrogen shortage. In this line, the shortage of electron transport by glucose addition during nitrogen deprivation induced a progressive decrease in the maximum Fd reduction (Figure S9).

Similar conclusions on the role of a reduced P700 in the bleaching process were reached on *Synechochoccus*, where promoting the oxidation of the PETC by adding nitrate (and thus consuming reduced Fd) prevented the induction of chlorosis and *nblA* expression caused by sub‐toxic MSX treatment (glutamine synthetase inhibitor) in the presence of ammonia (Klotz et al. 2015).

Together, our results demonstrate that, under nitrogen deprivation and glucose supplementation, the photosynthetic electron transport chain is disrupted at the PSII acceptor side (between Q_A_ and Q_B_). This leads to a lack of electron flow to and from PSI, indicating that, to proceed with bleaching, a certain threshold of reduced P700 is required to provide electrons to an unidentified molecular trigger. Therefore, these results highlight a redox‐controlled checkpoint in the nitrogen starvation response of *Synechocystis* and emphasize the tight metabolic‐photosynthetic control of photosynthetic organisms.

## Author Contributions

Pablo Ortega‐Martínez, Francisco J. Florencio, and Sandra Díaz‐Troya conceived the study together. Pablo Ortega‐Martínez carried out the experimental work, and was responsible for data analysis, visualization, and drafting the original manuscript. Joaquín Giner‐Lamia contributed to formal analysis and data visualization. Laura T. Wey provided essential resources and support for data analysis and visualization. M. Isabel Muro‐Pastor and Francisco J. Florencio oversaw project administration and secured funding. Sandra Díaz‐Troya supervised the project and contributed to data interpretation and visualization. All authors contributed to reviewing and editing the final manuscript.

## Conflicts of Interest

The authors declare no conflicts of interest.

## Supporting information


**Figure S1:** Supplementary Figures.

**Table S1:** ppl70645‐sup‐0002‐TableS1.xlsx.

**Table S2:** ppl70645‐sup‐0003‐TableS2.xlsx.

**Table S3:** ppl70645‐sup‐0004‐TableS3.xlsx.

**Table S4:** ppl70645‐sup‐0005‐TableS4.xlsx.

**Data S1:** Supplementary Method.

**Data S2:** Supporting Information.

## Data Availability

The RNA sequencing data used for Figure 3 (and its corresponding interactive Figure S1) and Tables [Link], [Link] are available in NCBI under accession number PRJNA1292945. Other data supporting the findings of this study are available in the manuscript or Supporting Information.
