# Supplementary material for: Glucose Uncouples Nitrogen Sensing From Chlorosis via a Photosynthetic Checkpoint in Synechocystis sp. PCC 6803
Source: Physiol Plant. 2025 Nov 21;177(6):e70645. doi: 10.1111/ppl.70645 (PMC12638213; doi:10.1111/ppl.70645)
Supplement: Supplementary file 1 — Figure S1: Supplementary Figures. [file PPL-177-e70645-s003.pdf]

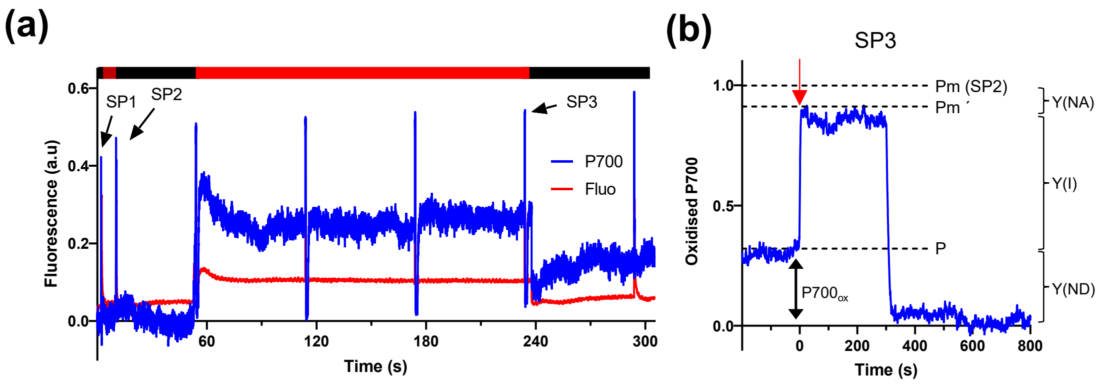

Figure S1. Example of a P700 redox kinetic.

(a) A representative induction curve (IC) in a Dual-PAM-100 for the simultaneous measurements of Chl fluorescence and redox kinetics of P700. The induction curve starts with a saturating pulse SP in darkness (SP1), where P700 is reduced. That is followed by the determination of maximum photooxidisable (Pm), achieved by a SP under far-red illumination (SP2), which preferentially excites PSI. This value is used in combination with the steady-state P700 oxidation level under actinic light (P) and maximum P700 oxidation level (Pm´) under a SP during actinic light (SP3) to calculate photosystem I quantum yields. (See figure 5). (b) Detail of a SP applied during actinic light (SP3) and description of parameters calculated, the effective quantum yield of PSI [Y(I)], and the non-photochemical energy dissipation due to donor-side [Y(ND)] and acceptor-side [Y(NA)] limitations. The red arrow denotes the onset of the 300 ms saturation pulse at 5000 µmol photons m^{-2} s^{-1}.

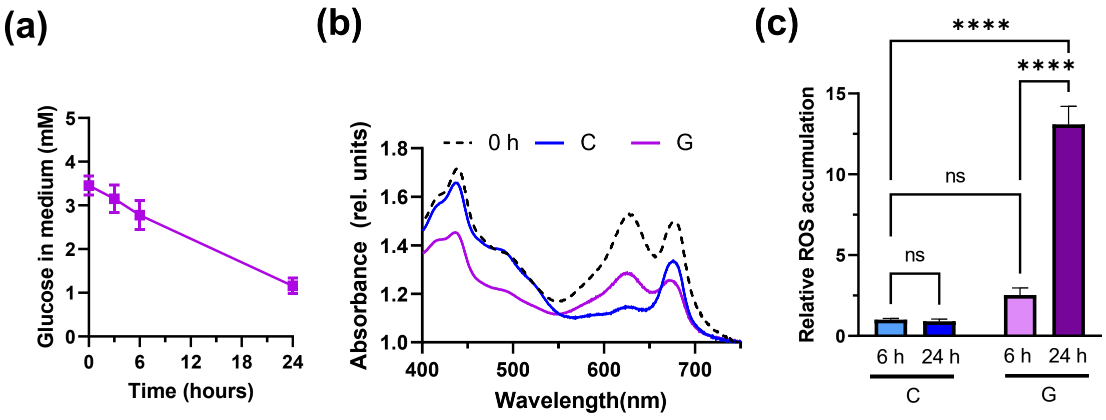

Figure S2. Consumption of glucose during nitrogen depletion and its effect on whole cell absorption profile, and reactive oxygen species production

WT cells grown in nitrogen-replete medium were harvested and resuspended in nitrogen-free ($BG11_0C$) medium at 1 OD_{750}. The culture was divided and grown under control (C) conditions or supplemented with 4 mM of glucose (G). (a) Glucose concentration profile in the media. (b) Representative whole cell spectra before (0 h) and 24 hours after culture under control conditions or supplemented with 4 mM of glucose. (c) Relative quantification of cellular reactive oxygen species (ROS) measured as 2',7'-Dichlorofluorescein fluorescence in cells cultured 6 and 24 hours in C and G conditions compared with levels of cells growing in nitrogen replete conditions. Data in (a) and (c) represent the mean±SEM of 4 independent biological replicates. Statistical significance was denoted as ****, P<0.0001; and ns, no significance (two-way ANOVA).

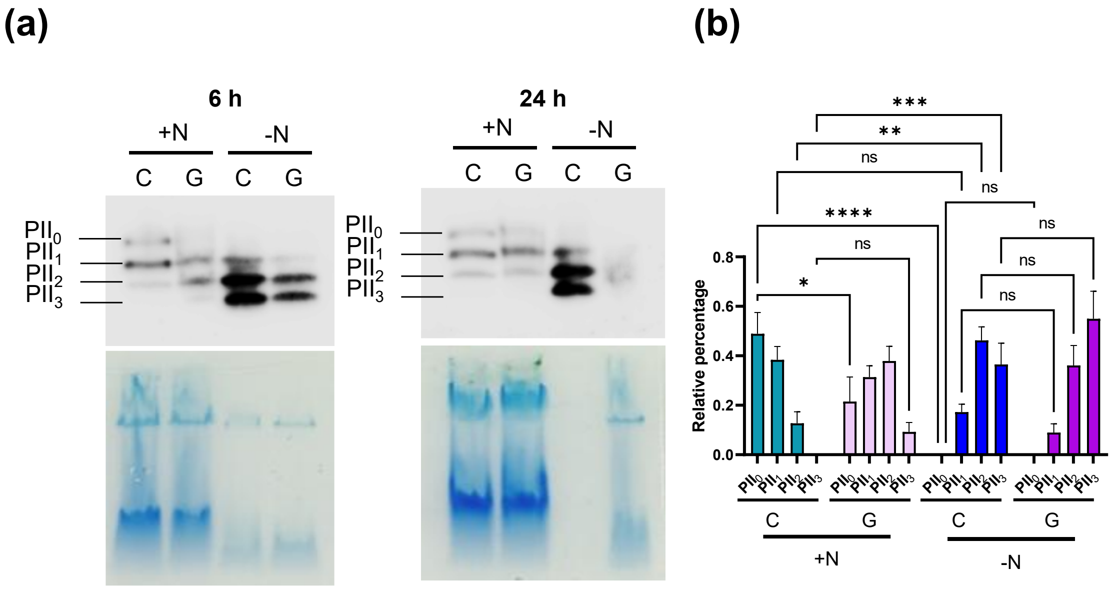

Figure S3. Phosphorilation state of PII in response to nitrogen deprivation and glucose supplementation

Native-PAGE analysis of soluble extracts of control (C) and glucose supplemented (G) cells after 6 and 24 hours in the presence (+N) or absence (-N) of nitrate. (a) Representative image of the immunochemoluminiscence against P-II protein (polyclonal antibody; 1:4000) and of the unstained polyacrylamide gels after electrophoresis of a total of 4 independent biological replicates is shown. (b) Quantification of phosphorylated PII bands after 6 hours of treatment. Data represent the mean±SEM of 4 independent biological replicates. Statistical significance was denoted as *, P<0.05; **, P<0.01; ***, P<0.001; ****, P<0.0001; ns, no significance (two-way ANOVA).

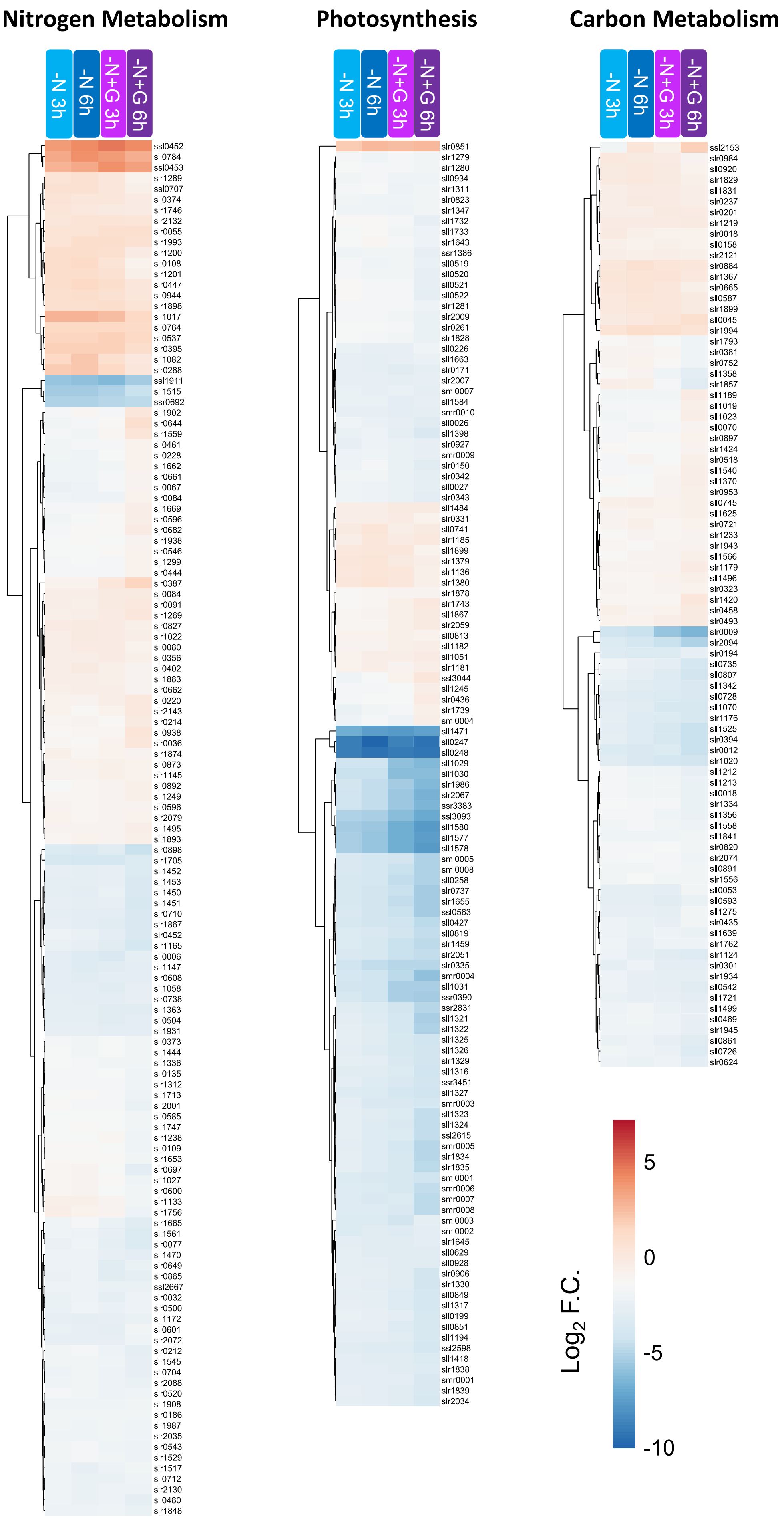

Nitrogen Metabolism
Photosynthesis
Carbon Metabolism
-N 3h
-N 6h
-N+G 3h
-N+G 6h
ssl0452 sll0784 ssl0453 slr1289 ssl0707 sll0374 slr1746 slr2132 slr0055 slr1993 slr1200 sll0108 slr1201 slr0447 sll0944 slr1898 slr1017 sll0764 sll0537 slr0395 slr1082 slr0288 ssl1911 sll1515 ssr0692 sll1902 slr0644 slr1559 sll0461 sll0228 slr1662 slr0661 sll0067 slr0084 sll1669 slr0596 slr0682 slr1938 slr0546 sll1299 slr0444 slr0387 sll0084 slr0091 slr1269 slr0827 slr1022 sll0080 sll0356 slr0402 sll1883 slr0662 sll0220 slr2143 slr0214 sll0938 slr0036 slr1874 sll0873 slr1145 sll0892 sll1249 sll0596 slr2079 sll1495 sll1893 slr0898 slr1705 sll1452 sll1453 sll1450 sll1451 slr0710 slr1867 slr0452 sll1165 sll0006 sll1147 slr0608 slr1058 slr0738 slr1363 sll0504 sll1931 sll0373 sll1444 sll1336 sll0135 slr1312 sll1713 sll2001 sll0585 sll1747 slr1238 sll0109 slr1653 slr0697 sll1027 slr0600 slr1133 slr1756 slr1665 sll1561 slr0077 sll1470 slr0649 slr0865 ssl2667 slr0032 slr0500 sll1172 sll0601 slr2072 slr0212 sll1545 sll0704 slr2088 slr0520 sll1908 slr0186 sll1987 slr2035 slr0543 slr1529 slr1517 sll0712 slr2130 sll0480 slr1848
slr0851 slr1279 slr1280 sll0934 slr1311 slr0823 slr1347 sll1732 sll1733 slr1643 ssr1386 sll0519 sll0520 sll0521 sll0522 slr1281 slr2009 slr0261 slr1828 sll0226 sll1663 slr0171 slr2007 sml0007 sll1584 smr0010 sll0026 sll1398 slr0927 smr0009 slr0150 slr0342 sll0027 slr0343 sll1484 slr0331 sll0741 slr1185 sll1899 slr1379 slr1136 slr1380 slr1878 slr1743 sll1867 slr2059 sll0813 sll1182 sll1051 slr1181 ssl3044 sll1245 slr0436 slr1739 sml0004 sll1471 sll0247 sll0248 sll1029 sll1030 slr1986 slr2067 ssr3383 ssl3093 sll1580 sll1577 sll1578 sml0005 sml0008 sll0258 slr0737 slr1655 ssl0563 sll0427 sll0819 slr1459 slr2051 slr0335 smr0004 sll1031 ssr0390 ssr2831 sll1321 sll1322 sll1325 sll1326 slr1329 sll1316 ssr3451 sll1327 smr0003 sll1323 sll1324 ssl2615 smr0005 slr1834 slr1835 sml0001 smr0006 smr0007 smr0008 sml0003 sml0002 slr1645 sll0629 sll0928 slr0906 slr1330 sll0849 sll1317 sll0199 sll0851 sll1194 ssl2598 sll1418 slr1838 smr0001 slr1839 slr2034
ssl2153 slr0984 sll0920 slr1829 sll1831 slr0237 slr0201 slr1219 slr0018 sll0158 slr2121 slr0884 slr1367 slr0665 sll0587 slr1899 sll0045 slr1994 slr1793 slr0381 slr0752 sll1358 slr1857 sll1189 sll1019 sll1023 sll0070 slr0897 slr1424 slr0518 sll1540 sll1370 slr0953 sll0745 sll1625 slr0721 slr1233 slr1943 sll1566 slr1179 sll1496 slr0323 slr1420 slr0458 slr0493 slr0009 slr2094 slr0194 sll0735 sll0807 sll1342 sll0728 sll1070 slr1176 sll1525 slr0394 slr0012 slr1020 sll1212 sll1213 sll0018 slr1334 sll1356 sll1558 sll1841 slr0820 slr2074 sll0891 slr1556 sll0053 sll0593 sll1275 slr0435 sll1639 slr1762 slr1124 slr0301 slr1934 sll0542 sll1721 sll1499 sll0469 slr1945 sll0861 sll0726 slr0624
Log₂ F.C.
5
0
-5
-10

Figure S4. Effect of glucose supplementation on the transcriptomic response of *Synechocystis* to nitrogen deprivation in genes related to carbon metabolism, photosynthesis, respiration, and nitrogen assimilation.

Heatmap showing the $\log_2$ fold-change of significantly differentially expressed genes (adj. *p-value* < 0.05) related to carbon metabolism, photosynthesis, respiration, and nitrogen assimilation after 3 and 6 hours under nitrogen deprivation (–N) and nitrogen deprivation with glucose supplementation (–N+G), compared to nitrogen-replete controls (0h). Comparisons shown: *–N 3h vs 0h, –N 6h vs 0h, –N+G 3h vs 0h, and –N+G 6h vs 0h*. Log_2 fold-change values are represented using a colour scale: red indicates upregulation and blue indicates downregulation.

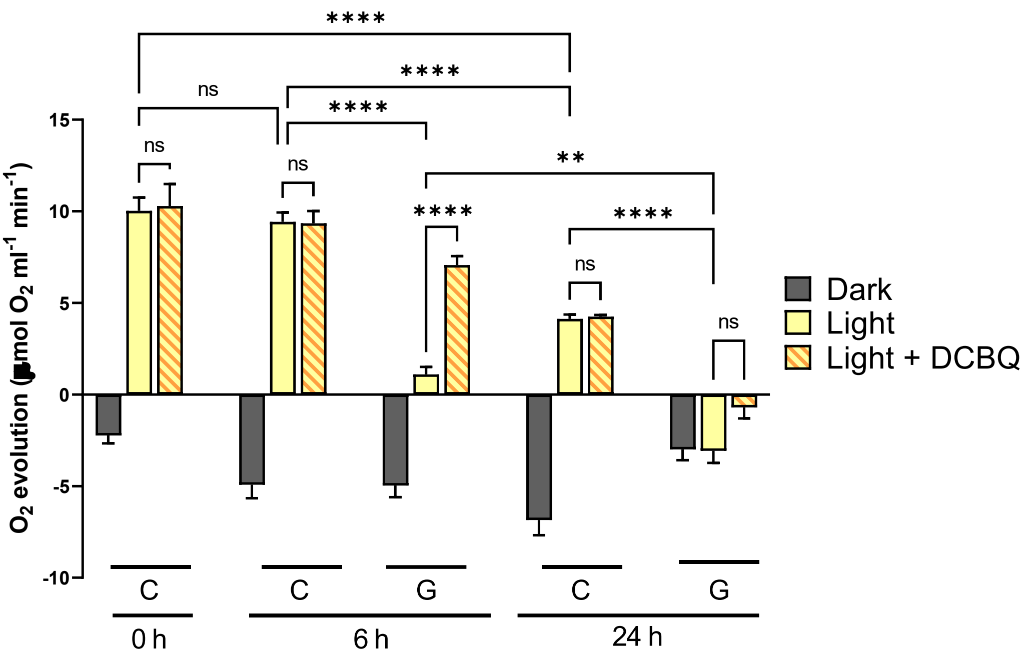

Figure S5. Effect of DCBQ on oxygen evolution during nitrogen depletion and glucose supplementation.

WT cells grown in nitrogen replete medium (BG11C) were harvested and resuspended in nitrogen-free medium ($BG11_0C$) at 1 OD_{750}. The culture was divided and grown under control (C) conditions or supplemented with 4 mM of glucose (G). The oxygen evolution rates of 2 mL of cultures at 6 and 24 hours after nitrogen deprivation were recorded on a Clark-type electrode with an illumination of 50 µmol photons m^{-2} s^{-1} and adding to the chamber a fresh mix of DCBQ (0,25 mM) and ferricyanide (2,5 mM). Data represent the mean±SEM of 4 independent biological replicates. Statistical significance was denoted as **, P<0.01; ****, P<0.0001; and ns, no significance (two-way ANOVA).

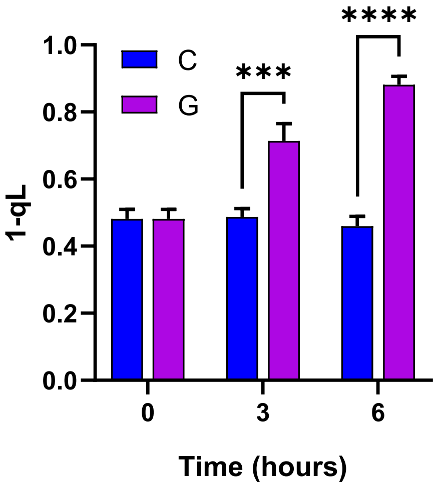

Figure S6: Chlorophyll fluorescence estimation of 1-qL during nitrogen depletion and glucose supplementation.

Estimations of 1-qL obtained from the traces in Figure 4. Data represent the mean±SEM of 5 independent biological replicates. Statistical significance was denoted as ***, $P < 0.001$ and ****, $P < 0.0001$ (two-way ANOVA).

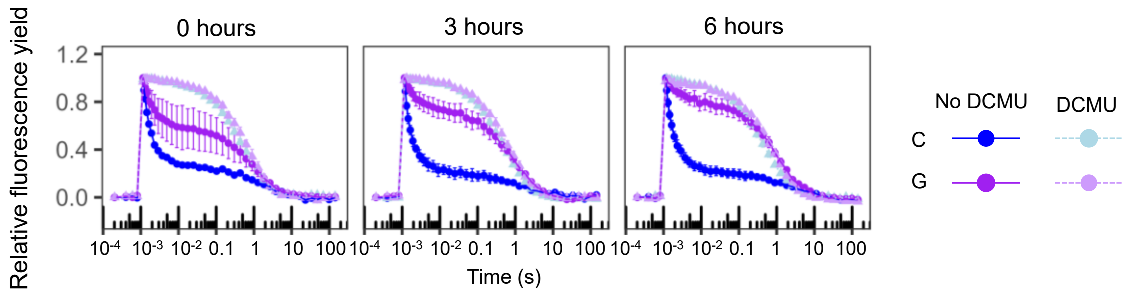

Figure S7: Q_A relaxation kinetics by chlorophyl fluorescence decay after a single turnover saturating pulse during nitrogen depletion and glucose supplementation.

WT cells grown in nitrogen-replete medium were harvested and resuspended in nitrogen-free ($BG11_0C$) medium at 1 OD_{750}. Reoxidation kinetics of Q_A^- with no addition (solid lines) or in the presence of 20 μM DCMU (dashed lines) of the cells grown under under control (C) conditions or supplemented with 4 mM of glucose (G) was performed at 0, 3 and 6 hours. Data was baselined to the fluorescence before the flash and normalized to the peak after the flash. Data represent the mean±SEM of 3 independent biological replicates.

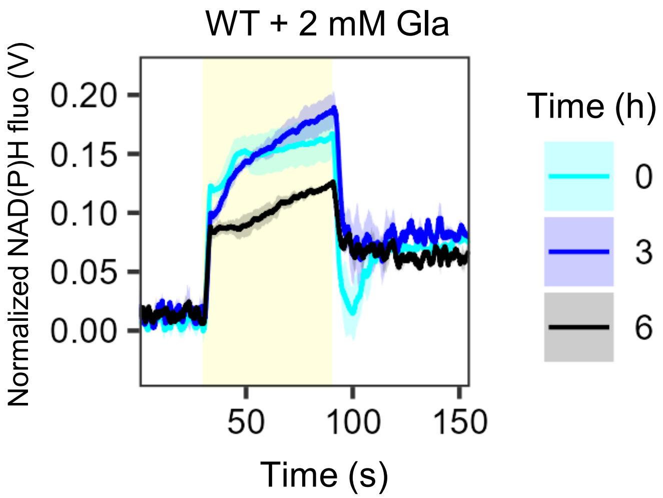

Figure S8. Effect of Gla treatment in NAD(P)H fluorescence light-dependent kinetics in *Synechocystis*

WT cells grown in nitrogen replete medium (BG11C) were harvested and resuspended in nitrogen-free medium ($BG11_0C$) at 1 OD_{750} with 2 mM of glycolaldehyde (Gla). NAD(P)H fluorescence light-dependent kinetics was registered at 0, 3 and 6 hours after nitrogen depletion and . Data represent the mean±SEM of 4 independent biological replicates.

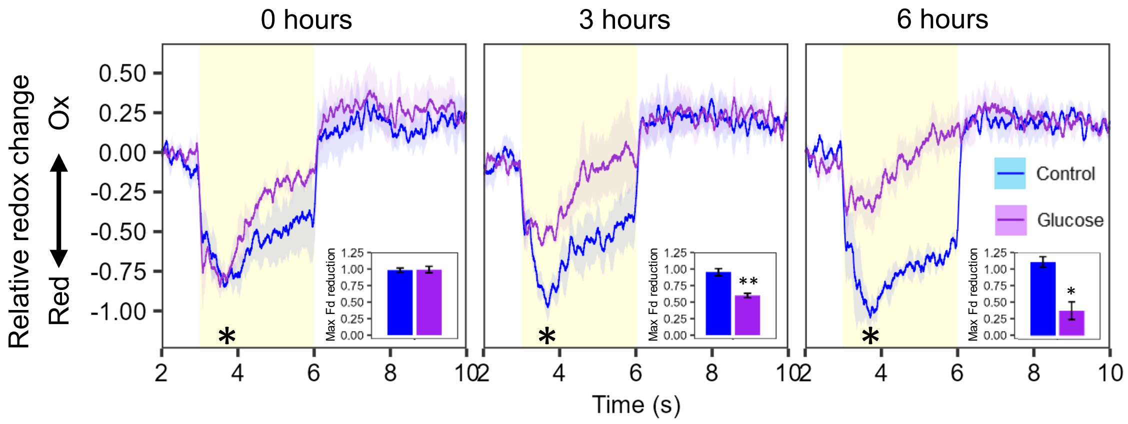

Figure S9. Ferredoxin redox characterization during nitrogen depletion and glucose supplementation.

WT cells grown in nitrogen-replete medium were harvested and resuspended in nitrogen-free ($BG11_0C$) medium at 1 OD_{750}. The cultures were adjusted to a concentration of 20 µg Chl ml^{-1} prior measurements. The relative redox change of the component Fd of the cells grown under under control (C) conditions or supplemented with 4 mM of glucose (G) was measured at 0, 3 and 6 hours in a DUAL-KLAS-NIR. Multiple turnover (MT) flashes indicated by an asterisk were provided following the light onset to fully reduce Fd. Yellow rectangles on the background represent a light exposure of 200 µmol photons m^{-2} s^{-1} intensity. Traces were normalized to the maximum Fd reduced value (corresponding to the MT flashes) and smoothed with a Savitzky–Golay filter (n = 99). Quantification of maximum Fd reduced values is provided as a bar graph inlet within each plot. Statistical significance was denoted as *, $P<0.05$; **, $P<0.01$; (two-tail t-test).

Data represent the mean±SEM of 3 independent biological replicates.
