# Supplementary material for: Glucose Uncouples Nitrogen Sensing From Chlorosis via a Photosynthetic Checkpoint in Synechocystis sp. PCC 6803
Source: Physiol Plant. 2025 Nov 21;177(6):e70645. doi: 10.1111/ppl.70645 (PMC12638213; doi:10.1111/ppl.70645)
Supplement: Supplementary file 6 — Data S1: Supplementary Method. [file PPL-177-e70645-s007.pdf]

Supplemental Material and methods

Protein extraction and immunoblotting

Fractions of 15 ml of cultures were harvested at six and 24 hours by centrifugation (5000 g 10 min 4º C) and storage frozen at -20º C. Frozen pellets were resuspended in 200 µl 50 mM Tris-HCl pH 7.5, 50 mM NaCl and 1 mM phenylmethylsulfonyl fluoride (PMFS) with glass beads (equivalent volume to 150-200 µL). Cell lysates were obtained after mechanical disruption by automatic disruption (at 6m/s for 30s in a FastPrep-24 5G; MP Biomedicals). Samples were centrifugated at 4 ºC for 20 min at 15000 g and 150 µl of supernatant, containing soluble proteins, was collected in a new tube and mixed with 50 µl of loading buffer without reducing agents or SDS. In general, 20 µl of each sample was resolved by Native-PAGE (15% acrylamide/bis-acrylamide). For –N at six hours samples and for C –N at 24 hours, 5- and 10-fold dilutions were loaded. After electrophoresis, proteins were transferred to a polyvinylidene difluoride membrane (Immobilon-P; Millipore). Blocking, probing and detection was performed as in (Ortega-Martínez et al. 2023) using primary antibody against P-II protein (1:4000) diluted in blocking solution.

Reactive oxygen species measurement

Reactive oxygen species levels were measured in (Ortega-Martínez et al. 2024). Cellular pellets from 1 ml culture (centrifuged at 10 000 g for 5 min) were resuspended in 0.8 ml of 1 x phosphate-buffered saline (PBS) containing 50 µM of the fluorescent probe 2´,7´-dichlorofluorescein diacetate (DCFH-DA, D6883 Sigma-Aldrich). Samples were incubated for 1 h in darkness at 30°C with agitation. After incubation, samples were centrifuged at 10 000 g for 5 min and washed once with 1 x PBS to wash free DCFH-DA. Finally, cells were resuspended in 200 µl PBS and transferred to a black-walled, clear-bottom, 96-well microplate. Fluorescence of dichlorofluorescein (DCF; the oxidised form of DCFH), was measured at 525 nm after excitation at 488 nm in a Varioskan multiplate reader.

Q_A^- reoxidation kinetics

The kinetics of the chlorophyll fluorescence decay after a single-turnover saturating flash was performed similarly as in (Ortega-Martínez et al. 2024). Chl

fluorescence of cultures at 5 µg Chl ml^{-1} and dark-adapted for 5 min was monitored after a single-turnover flash using an FL 3500 fluorometer (PSI Instruments, Drásov, Czech Republic). Same sample was subsequently measured after adding 10 µM 3-(3,4-dichlorophenyl)-1,1-dimethylurea (DCMU). Each trace was baselined to the fluorescence before the flash and normalised to the maximum fluorescence after the flash.

Deconvolution of redox changes of Fd

The deconvolution of redox changes of Fd was performed as in (Ortega-Martínez et al. 2024) using a DUAL-KLAS-NIR spectrophotometer (Walz) and a NIRMAX script (Schreiber and Klughammer 2016) that was modified for cyanobacteria (3 s AL with a MT at 200 ms to obtain maximal values of Fd reduction). The model spectra for deconvolution of *Synechocystis* PC, P700, and Fd signals were the ones obtained in (Nikkanen et al. 2020).

REFERENCES:

Nikkanen L, Santana Sánchez A, Ermakova M, Rögner M, Cournac L, Allahverdiyeva Y (2020) Functional redundancy between flavodiiron proteins and NDH-1 in Synechocystis sp. PCC 6803. Plant Journal 103: 1460–1476

Ortega-Martínez P, Nikkanen L, Wey LT, Florencio FJ, Allahverdiyeva Y, Díaz-Troya S (2024) Glycogen synthesis prevents metabolic imbalance and disruption of photosynthetic electron transport from photosystem IIduring transition to photomixotrophy in Synechocystis sp. PCC6803. New Phytologist 243:

Ortega-Martínez P, Roldán M, Díaz-Troya S, Florencio FJ (2023) Stress response requires an efficient connection between glycogen and central carbon metabolism by phosphoglucomutases in cyanobacteria. J Exp Bot 74: 1532–1550

Schreiber U, Klughammer C (2016) Analysis of photosystem I donor and acceptor sides with a new type of online-deconvoluting kinetic LED-array spectrophotometer. Plant Cell Physiol 57: 1454–1467
